# Improved psychosocial measures associated with physical activity may be explained by alterations in brain-gut microbiome signatures

**DOI:** 10.1038/s41598-023-37009-z

**Published:** 2023-06-26

**Authors:** Michelle Guan, Tien S. Dong, Vishvak Subramanyam, Yiming Guo, Ravi R. Bhatt, Allison Vaughan, Robert L. Barry, Arpana Gupta

**Affiliations:** 1 David Geffen School of Medicine, Los Angeles, USA; 2 Vatche and Tamar Manoukian Division of Digestive Diseases, Los Angeles, USA; 3 Goodman-Luskin Microbiome Center at UCLA, Los Angeles, USA; 4 University of California, Los Angeles, USA; 5 Division of Gastroenterology, Hepatology and Parenteral Nutrition, VA Greater Los Angeles Healthcare System, Los Angeles, CA USA; 6 G. Oppenheimer Center for Neurobiology of Stress and Resilience, Los Angeles, USA; 7 Imaging Genetics Center, Mark and Mary Stevens Institute for Neuroimaging and Informatics, Keck School of Medicine at USC, University of Southern California, Los Angeles, USA; 8 Athinoula A. Martinos Center for Biomedical Imaging, Department of Radiology, Massachusetts General Hospital, Charlestown, MA USA; 9 Department of Radiology, Harvard Medical School, Boston, MA USA; 10 Harvard-Massachusetts Institute of Technology Health Sciences & Technology, Cambridge, MA USA

**Keywords:** Stress and resilience, Obesity, Psychiatric disorders, Obesity

## Abstract

Obesity contributes to physical comorbidities and mental health consequences. We explored whether physical activity could influence more than metabolic regulation and result in psychological benefits through the brain-gut microbiome (BGM) system in a population with high BMI. Fecal samples were obtained for 16 s rRNA profiling and fecal metabolomics, along with psychological and physical activity questionnaires. Whole brain resting-state functional MRI was acquired, and brain connectivity metrics were calculated. Higher physical activity was significantly associated with increased connectivity in inhibitory appetite control brain regions, while lower physical activity was associated with increased emotional regulation network connections. Higher physical activity was also associated with microbiome and metabolite signatures protective towards mental health and metabolic derangements. The greater resilience and coping, and lower levels of food addiction seen with higher physical activity, may be explained by BGM system differences. These novel findings provide an emphasis on the psychological and resilience benefits of physical activity, beyond metabolic regulation and these influences seem to be related to BGM interactions.

## Introduction

In the past few decades, obesity rates have rapidly grown to epidemic proportions, with an estimated 650 million adults considered obese^1^. High BMI leads not only to the development of physical comorbidities, but also to mental health consequences, in a bidirectional relationship that likely explains the high comorbidity that is seen between obesity and the development of psychiatric disorders^2^. Individuals who have reported weight-related discrimination are more likely to engage in high-risk behaviors such as drug abuse and cigarette smoking, and also tend to experience greater weight gain over time^3^. Additionally, there is a greater likelihood of developing maladaptive eating behaviors, including food addiction, binge-eating, emotional eating, and increased consumption of calories^4,5^.

In individuals with higher BMI, modifiable factors such as resilience may serve a protective function against the predisposition of developing psychiatric disorders with obesity^6^. Resilience is defined as the ability to positively adapt in response to significant adversity or stressors, and develops via interplay between genetics, environmental factors, and social support systems^7^. Studies have shown that emotional resilience is protective against the development of obesity regardless of income, through positive associations with healthier dietary choices and moderating perceived stress and binge eating behavior^8,9^. In adults, physical activity (PA) is a well-recognized contributor to psychological resilience by blunting stress reactivity, protecting against the metabolic consequences of stress-inducing events, and promoting an anti-inflammatory state^10–12^. While some studies have been done on the individual physiological changes associated with PA in high BMI populations, there are a limited number of studies on the interactions between PA and various psychological variables in the context of the brain-gut microbiome (BGM) system.

**Figure 1:**
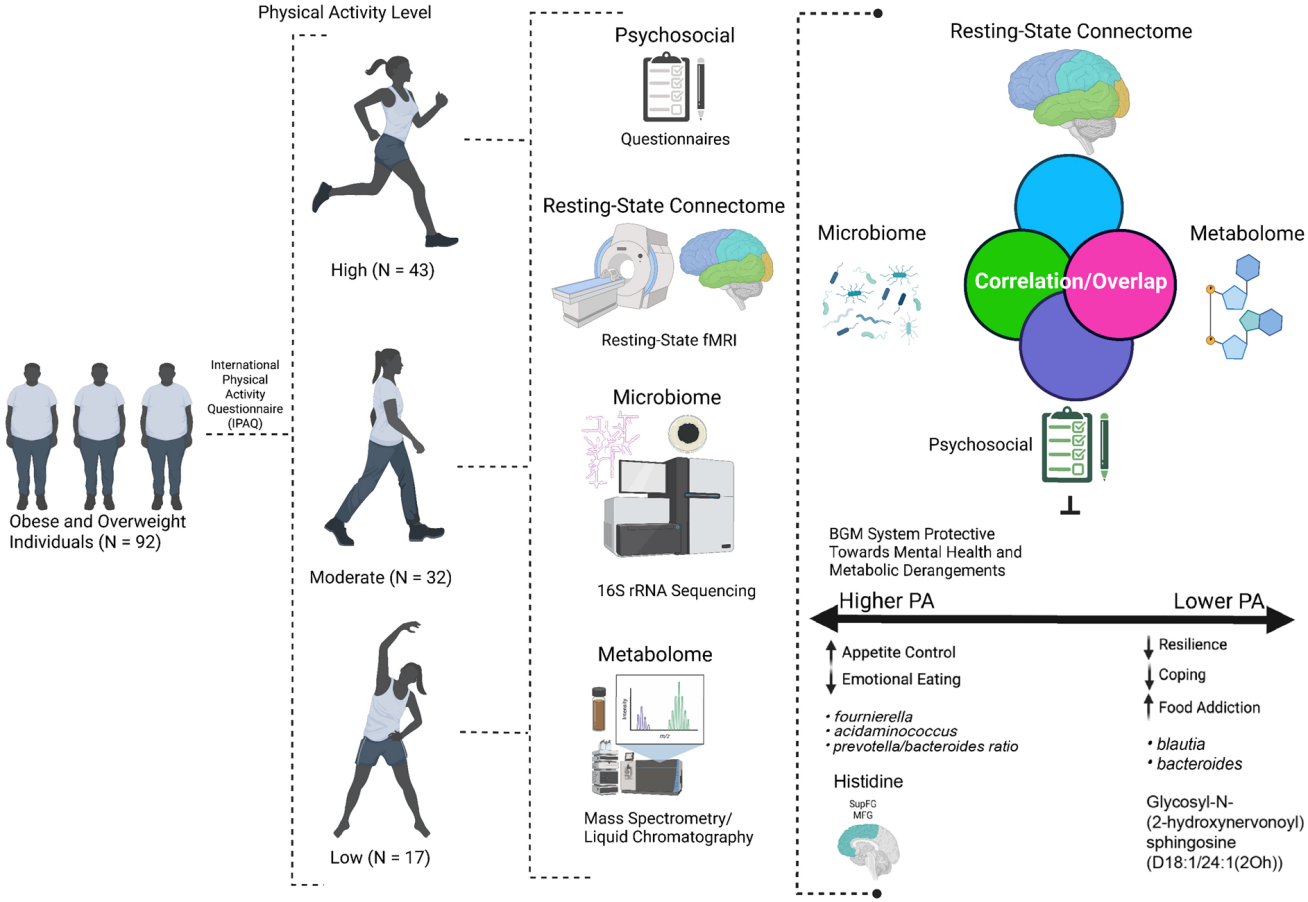
Study Overview, Workflow, and Results. Denotes the overview of workflow and analyses, and summarizes the findings from the study.

A growing body of studies support the role of the BGM axis in the pathophysiology of obesity, mediated by alterations in metabolic, enteroendocrine, and neural signaling^13^. Having high BMI has been associated with changes in the diversity and composition of the gut microbiota, which lead to disruptions in the downstream metabolites and gut-endocrine signals that orchestrate energy homeostasis^14^. For example, obesity was found to be associated with increases in the Prevotella/Bacteroides ratio and decreases in fecal tryptophan levels, which is a metabolite related to the biosynthesis of serotonin^15^. Signals from the microbiome may thus also alter neural processes, with individuals with high BMI demonstrating alterations in reward and emotional regulation brain regions, which have also been linked to clinical measures such as food addiction, involving continued consumption of palatable foods despite meeting homeostatic energy requirements^16,17^. While the effects of PA on the brain, microbiome, and metabolites may have been examined independently, there is a lack of studies that utilize a systems-biology approach to study the effects of PA within the BGM as an integrated system, while incorporating psychosocial variables in the context of obesity. In this study, we predict that there are distinct brain, gut microbiome, and metabolite signatures based on PA, and that these BGM system associations modulate positive psychological changes in a population with high BMI (summarized in Fig. 1).

## Methods

The sample recruited was comprised of 92 participants, with the absence of significant medical or psychiatric conditions. All procedures were performed in accordance with the relevant guidelines and regulations and were approved by the Institutional Review Board (16-000187, 16-000281) at the University of California, Los Angeles's Office of Protection for Research Subjects. All participants provided written informed consent.

### Participants

Participants were selected as described in our previous studies^15^. Participants were excluded for the following: pregnant or lactating, substance use, abdominal surgery, tobacco dependence (half a pack or more daily), extreme strenuous exercise (> 8 h of continuous exercise per week such as marathon runners), current or past psychiatric illness and major medical or neurological conditions. Participants taking medications that interfere with the central nervous system or regular use of analgesic drugs were excluded. Because of the effect of handedness on fMRI activation, only right-handed participants were included to negate handedness as a cofounder. To avoid potential cofounders in microbiome analysis, included participants were also required to not have taken antibiotics or probiotics for at least 3 months before enrolling in the study. Only premenopausal females were enrolled and were scanned during the follicular phase of their menstrual cycles as determined by self-report of their last day of the cycle. Participants with hypertension, diabetes, metabolic syndrome or eating disorders were excluded to minimize confounding effects. We used body mass index (BMI) cutoffs to define our overweight (25 ≤ BMI < 30) and obese (BMI ≤ 30) groups. No participants exceeded 400lbs due to magnetic resonance imaging (MRI) scanning weight limits. Participants underwent MRI scans, anthropometrics (height, body weight, and body mass index), and fresh stool samples for 16 s ribosomal RNA gene sequencing and metabolite analysis were collected.

### Questionnaires

Various questionnaires were utilized to assess participant’s physical activity levels and psychological well-being. Participants completed the validated International Physical Activity Questionnaire (IPAQ) long form, which comprises of 27 items that collected data in different domains (job-related, transport-related, domestic and leisure-time physical activity) and intensities (moderate, vigorous, walking) and includes sitting time^18^. The Guidelines for Data Processing and Analysis of the IPAQ categorical scoring were used to determine participants’ current level of physical activity and participants were grouped into low, moderate, or high physical activity level categories^19^.

Additionally, psychological resiliency was assessed using the Brief Resilience Scale (BRS)^20^. To assess stress, anxiety, and mood, the Hospital Anxiety and Depression Scale (HADS) was used. The HADS is a 14-item scale used to measure symptoms of anxiety and depression^21^. The questions are scored on a scale of 0–3, corresponding to how much the individual identifies with the question for the past week. In order to assess effective and ineffective coping strategies, participants also completed the Brief-COPE questionnaire, which is a 28 item self-report questionnaire that comprises of 14 two-item subscales including: (1) self-distraction, (2) active coping, (3) denial, (4) substance use, (5) use of emotional support, (6) use of instrumental support, (7) behavioral disengagement, (8) venting, (9) positive reframing, (10) planning, (11) humor, (12) acceptance, (13) religion, and (14) self-blame^22^.

Food addiction was assessed using the Yale Food Addiction Scale (YFAS) questionnaire, a 25-item scale developed to assess food addiction by assessing signs of substance-dependence symptoms in eating behavior^23^. This scale is based upon the substance dependence criteria as found in the DSM (e.g. tolerance [marked increase in amount; marked decrease in effect], withdrawal [agitation, anxiety, physical symptoms], and loss of control [eating to the point of feeling physical ill])^23^. The YFAS questionnaire is a 25-question survey that measures several aspects of food addiction behavior: food dependence, withdrawal, tolerance, continued use despite problems, time spent eating, loss of control, inability to cut down, and clinically significant impairment. Food addiction was defined as having a YFAS symptom count ≥ 3 with clinically significant impairment or distress. Clinically significant impairment or distress was defined as having a at least one positive response to the following two questions in the YFAS questionnaire: “My behavior with respect to food and eating causes significant distress” and “I experience significant problems in my ability to function effectively (daily routine, job/school, social activities, family activities, health difficulties) because of food and eating,” similar to previously published works^24^. The YFAS has displayed a good internal reliability (Kuder–Richardson *α* = 0.86)^23^.

All patients underwent the UCLA Diet Checklist, which is a questionnaire developed by our institution, intended to represent the diet that best reflects what participants consume on a regular basis, and has been used in our previously published works^25^. The specific diets incorporated into this checklist were: Standard American (characterized by high consumption of processed, frozen, and packaged foods, pasta and breads, and red meat; vegetables and fruits are not consumed in large quantities), Modified American (high consumption of whole grains including some processed, frozen, and packaged foods; red meat is consumed in limited quantities; vegetables and fruit are consumed in moderate to large quantities), Mediterranean (high consumption of fruits, vegetables, beans, nuts, and seeds; olive oil is the key monounsaturated fat source; dairy products, fish, and poultry are consumed in low to moderate amounts and little red meat is eaten), and all other diets that do not fit into the above categories (vegan, vegetarian, and gluten-free). This Diet Checklist was then internally validated against the standardized DHQ-III. For data analysis, we combined standard American and modified American diet as one category. Mediterranean, and all other diets were combined as “other” for analysis.

### Microbiome: DNA extraction, 16S sequencing, alpha/beta diversity analyses, differential abundance testing

DNA from stool was extracted using the DNA Fecal Microbe Miniprep Kit (Zymo Research). The V4 region of 16S ribosomal RNA was amplified and underwent paired end sequencing on an Illumina HiSeq 2500 (San Diego, CA, USA) as previously described^26^. Sequences were processed through the DADA2 pipeline to generate exact amplicon sequence variants (ASVs) and taxonomy was assigned based upon the SILVA 138 database^27^. Microbial alpha diversity was assessed on data rarefied to equal sequencing depth applying metrics including the Chao1 index of richness and the Shannon index of evenness. Microbial composition (i.e. beta diversity) was compared across groups using robust Aitchison (a phylo-genetic distance metric) in QIIME2 and visualized with principal coordinates analysis^28,29^. The significance of beta diversity, adjusting for covariates, was assessed using multivariate PERMANOVA with significance determined by 100,000 permutations^30^. Predicted metagenomics was performed using PICRUSt2 in QIIME2 using the default settings to predict abundances of bacterial gene families annotated as KEGG orthologs (KO) based on nearest reference genomes to 16S sequences.

Differential abundance of microbes was analyzed using MaAslin2, which utilizes a generalized linear mixed model with total sum scaling normalization for microbiome data^32^. Predicted metagenome differences between groups was visualized through principal component analysis (PCA) and significance tested using PERMANOVA. Individual predicted genes were tested between groups using DESEq2 in R and corrected for multiple hypothesis testing using false discovery rate (FDR) correction (q < 0.05 for significance). The raw sequences can be accessed NIH NCBI BioProject (BioProject ID: PRJNA946906).

### Metabolites

Using the same fecal samples as the 16S sequencing, samples were aliquoted under liquid nitrogen and then shipped to Metabolon. They were processed and analyzed as a single batch on Metabolon’s global metabolomics and bioinformatics platform. Using established protocols, data was curated by mass spectrometry as previously reported^33^. An untargeted metabolomics platform was used, and values were scaled and relative.

### Brain: MRI acquisition

Whole brain structural and resting state functional connectivity data was collected using a 3.0 T Siemens Prisma MRI scanner (Siemens, Erlangen, Germany). Detailed information on the standardized acquisition protocols, quality control measures, and image preprocessing are provided in previously published studies^15,25^.

For the structural MRI acquisition, high resolution T1-weighted images were acquired: echo time/repetition time (TE/TR) = 3.26 ms/2200 ms, field of view = 220 × 220 mm, slice thickness = 1 mm, 176 slices, 256 × 256 voxel matrix, and voxel size = 0.86 × 0.86 × 1 mm^25^.

Whole brain resting state scans were acquired with eyes closed and an echo planar sequence with the following parameters: TE/TR = 28 ms/2000 ms, flip angle = 77°, scan duration = 10 m6s, FOV = 220 mm, slices = 40, and slice thickness = 4.0 mm^25^.

### Brain: functional network construction

Functional brain networks were constructed as previously described^15,34^. To summarize, measures of region-to-region functional connectivity (Fisher transformed Pearson’s correlations) were computed using the CONN toolbox and the aCompCor method in Matlab Confounding factors such as white matter, cerebrospinal fluid, the six motion realignment parameters, and the root mean squared values of the detrended realignment estimates were regressed out for each voxel using ordinary least squares regression on the normalized, smoothed resting-state images^35^. Participants with RMS values over 0.25 were not included. Images were then filtered using a band-pass filter (0.008/s < f < 0.08/s) to reduce the low and high-frequency noises. Although the influence of head motion cannot be completely removed, CompCor has been shown to be particularly effective for dealing with residual motion relative to other methods^31^. Regions of interest were segmented with the Harvard–Oxford Subcortical atlases, the Schaefer 400 cortical atlas, and the Ascending Arousal Network brainstem atlas^36,37^. These atlases parceled into a total of 430 brain regions. The ROI-ROI functional connectivity between the brain regions was indexed by a matrix of Fisher Z transformed correlation coefficients reflecting the association between average temporal BOLD time series signals across all voxels in each brain region. The magnitude of the Z value represents the weights in the functional network. Permuted statistical values from ROI-to-ROI analyses were further corrected using the false discovery rate (FDR) to measure significance with p(FDR) < 0.05.

### Statistical analysis

General linear model (GLM) with linear contrasts were applied to examine group differences in baseline demographic and behavioral differences (High PA vs. Low PA, High PA vs. Moderate PA, Moderate PA vs. Low PA). Means were reported with their corresponding standard deviations.

We calculated beta diversity using DEICODE plugin in QIIME 2, which accounts for sparse compositional nature of microbiome data with a robust Aitchison analysis. This method has been shown to yield higher discriminatory power compared to other common metrics, such as UniFrac or Bray-Curtis^28^. Alpha diversity was calculated in QIIME 2 using data rarefied to 32,303 sequences and significance was determined using Chao1 and Shannon index by analysis of variance. Association of microbial genera were evaluated using MaAslin2 in R, which uses a generalized linear mixed model with total sum scaling normalization. Differential abundance p-values were converted to q-values to adjust for multiple hypothesis testing using a false discovery rate (FDR) correction (q < 0.05 for significance).

Sparse partial least squares discriminant analysis (sPLS-DA) was conducted using the R package mixOmics as a data reduction method for the resting-state brain connectivity and metabolites separately as previously described^38,39^. Prior to analyses, resting-state connectivity and the metabolite datasets were preprocessed. The identification of near zero variance predictors was determined on the metabolite data and then removed with the cutoff being 50% of the values must be distinct with respect to the number of subjects. The resting-state functional connectivity and metabolomics data were scaled and centered.

For integrated analyses, significant findings from fMRI, metabolite, 16S microbiome, and clinical data were combined into one dataset, and Spearman’s correlations between datapoints were performed using the *Hmisc* and *corrplot* packages in R. All p-values were adjusted for multiple hypothesis testing using (FDR) correction (q < 0.05 for significance). A summary of the workflow can be visualized in Fig. 1.

## Results

### Participant characteristics and psychosocial measures

Psychosocial and behavioral characteristics of the 92 individuals (males = 24, females = 68) who are overweight or obese (mean BMI = 33.22 kg/m^2^, mean age = 32.84 years) are summarized in Table 1. Based on the IPAQ scoring guidelines for determining PA levels, the average total PA in the high (n = 43, males = 15, females = 28), moderate (n = 32, males = 5, females = 27), and low (n = 17, males = 4, females = 13) groups were 13,432.84 MET minutes, 5,081.70 MET minutes, and 1822.953 MET minutes respectively (p < 0.001). There were no significant differences in education or income levels between the groups, except within the high versus moderate PA comparison for education level (p = 0.05).

**Table 1:** Participant’s psychosocial characteristics Based on physical Activity Levels.

	All (N = 92)				High PA (N = 43)			Moderate PA (N = 32)			Low PA (N = 17)			High vs Mod PA	Mod vs. Low PA	High vs. Low PA
	Mean	SD	Range	N	Mean	SD	Range	Mean	SD	Range	Mean	SD	Range	p-value		
Age	32.84	10.30	[18, 54]	92	35.12	11.77	[18, 54]	29.65	8.67	[19, 54]	31.47	8.12	[19, 53]	0.14	0.48	0.08
BMI	33.22	4.54	[25.32, 47.54]	92	33.25	4.50	[25.59, 45.29]	33.98	4.33	[25.32, 42.07]	32.77	5.19	[27.27, 47.54]	0.65	0.39	0.59
Education and socioeconomic status																
Education	4.81	0.94	[2, 6]	89	4.60	0.94	[2, 6]	4.94	0.89	[3, 6]	5.03	0.97	[3, 6]	0.05	0.74	0.21
Income	6.55	2.46	[1, 9]	89	6.46	2.67	[1, 9]	6.73	2.07	[1, 9]	6.41	2.69	[1, 9]	0.89	0.90	1.00
Physical activity (IPAQ)																
Total walking	3935.06	4225.91	[0, 26,099.41]	92	5952.78	4801.98	[609, 26,099.41)	2725.83	1189.69	[0, 4223.71)	1107.60	2985.03	[0, 14157]	1.29E-03	0.04	1.34E-04
Total moderate	2508.86	3524.13	[0, 21840]	92	3870.01	4086.79	[0, 21840]	1769.37	425.30	[0, 1260]	457.94	2880.03	[0, 14669]	0.02	0.07	1.16E-03
Total vigorous	1938.87	4232.45	[0, 32688]	92	3610.05	5685.29	[0, 32688]	586.50	644.67	[0, 2576]	257.41	1125.42	[0, 5440]	4.14E-03	0.27	0.02
Total physical activity	8382.79	8936.20	[0, 56,507.11]	92	13,432.84	9488.34	[3834, 56,507.11)	5081.70	1824.55	[0, 7516.71)	1822.95	6364.02	[772, 34266]	5.09E-05	0.05	5.95E-06
Resilience																
BRS score	22.681	4.718	[9, 30]	91	23.9286	4.8760	[9, 30]	21.906	3.913	[16, 29]	21.059	4.596	[13, 30]	0.07	0.52	0.04
Brief cope																
Self distraction	4.70	1.70	[2, 8]	88	4.56	1.66	[2, 8]	5.03	1.70	[2, 7]	4.47	1.75	[2, 8]	0.25	0.29	0.85
Active coping	5.63	1.91	[2, 8]	88	5.78	1.98	[2, 8]	5.77	1.73	[2, 8]	5.00	1.91	[2, 8]	0.98	0.18	0.16
Denial	2.38	0.93	[2, 8]	87	2.25	0.67	[2, 5]	2.53	0.87	[2, 5]	2.41	1.22	[2, 8]	0.22	0.72	0.45
Substance use	2.20	0.79	[2, 8]	88	2.29	1.05	[2, 8]	2.13	0.49	[2, 8]	2.12	0.43	[2, 4]	0.44	0.91	0.52
Emotional support	5.52	2.01	[2, 8]	88	5.54	2.12	[2, 8]	5.70	1.91	[2, 8]	5.18	1.95	[2, 8]	0.74	0.38	0.55
Intrumental support	5.41	2.07	[2, 8]	88	5.46	2.23	[2, 8]	5.77	1.80	[2, 8]	4.65	1.94	[2, 8]	0.55	0.06	0.19
Behavioral disengagemnet	2.42	0.74	[2, 5]	88	2.37	0.70	[2, 4]	2.53	0.61	[2, 8]	2.35	0.86	[2, 5]	0.37	0.45	0.95
Venting	4.20	1.68	[2, 8]	87	4.07	1.63	[2, 8]	4.27	1.75	[2, 7]	4.38	1.76	[2, 8]	0.63	0.84	0.54
Positive reframing	5.28	1.94	[2, 8]	88	5.46	1.92	[2, 8]	5.47	1.70	[2, 7]	4.53	2.03	[2, 8]	0.99	0.11	0.09
Planning	5.84	1.73	[2, 8]	89	6.02	1.77	[2, 8]	5.97	1.81	[2, 8]	5.18	1.59	[2, 8]	0.89	0.13	0.10
Humor	4.30	1.77	[2, 8]	89	4.43	1.85	[2, 8]	4.43	1.56	[2, 6]	3.76	1.77	[2, 8]	0.99	0.20	0.20
Acceptance	5.72	1.81	[2, 8]	89	6.17	1.82	[2, 8]	5.47	1.71	[2, 8]	5.06	1.74	[2, 8]	0.11	0.44	0.04
Religion	4.51	2.20	[2, 8]	87	4.73	2.26	[2, 8]	4.68	1.85	[2, 8]	3.53	2.23	[2, 8]	0.92	0.09	0.07
Self blame	3.83	1.70	[2, 8]	89	3.95	1.86	[2, 8]	3.73	1.57	[2, 8]	3.71	1.57	[2, 8]	0.60	0.95	0.63
Stress and anxiety																
HAD anxiety	5.12	3.71	[0, 14]	92	4.56	3.82	[0, 14]	5.84	3.56	[1, 13]	5.18	3.61	[0, 14]	0.14	0.54	0.57
HAD depression	2.46	2.60	[0, 13]	92	1.95	2.37	[0, 13]	2.81	3.11	[0, 10]	3.06	2.56	[0, 10]	0.14	0.77	0.14
Food cravings (Yale Food Addiction Scale)																
Withdrawal	0.217	0.531	[0, 3]	92	0.16	0.43	[0, 2]	0.19	0.87	[0, 3]	0.41	0.40	[0, 1]	0.80	0.22	0.14
Tolerance	0.205	0.529	[0, 2]	88	0.20	0.51	[0, 2]	0.10	0.73	[0, 2]	0.44	0.40	[0, 2]	0.38	0.04	0.16
Continued use	0.239	0.429	[0, 1]	92	0.19	0.39	[0, 1]	0.47	0.51	[0, 1]	0.19	0.40	[0, 1]	0.99	0.04	0.02
Given up	0.165	0.601	[0, 4]	91	0.05	0.22	[0, 1]	0.09	1.18	[0, 4]	0.59	0.39	[0, 2]	0.52	0.03	0.01
Time spent	0.304	0.588	[0, 3]	92	0.16	0.43	[0, 2]	0.22	0.88	[0, 3]	0.82	0.42	[0, 1]	0.58	2.07E-03	0.00
Loss control	0.110	0.379	[0, 2]	91	0.05	0.31	[0, 2]	0.06	0.61	[0, 2]	0.35	0.25	[0, 1]	0.82	0.02	0.01
Unsuccessful cut down	1.716	0.909	[0, 4]	88	1.64	1.01	[0, 4]	1.72	0.86	[1, 4]	1.88	0.80	[1, 4]	0.72	0.53	0.39
ClinSig impairment	0.120	0.415	[0, 2]	92	0.05	0.21	[0, 1]	0.16	0.56	[0, 2]	0.24	0.51	[0, 2]	0.21	0.62	0.06
Symptom count	1.924	1.477	[0, 7]	92	1.60	1.12	[0, 5]	1.69	1.98	[1, 7]	3.18	1.28	[0, 6]	0.77	2.50E-03	2.52E-04
Body mass (Bioimpedance analysis)																
Fat mass (%)	35.42	8.09	[3, 51.10]	90	34.10	8.78	[3, 50.10)	36.43	8.56	(14.10, 47.60)	36.84	6.73	(20.29, 51.10)	0.22	0.86	0.28
Lean body mass (%)	64.57	8.09	[48.90, 97]	90	65.88	8.77	(49.90, 97]	63.57	8.56	(52.40, 85.91)	63.17	6.73	(48.90, 79.70)	0.23	0.86	0.28
Total weight (%)	100.00	0.00	[100, 100]	78	100.00	0.00	[100, 100]	100.00	0.00	[100, 100]	100.00	0.00	[100, 100]	0.42	0.45	0.54

Means and standard deviations are reported for normally distributed data. P-significant < 0.05.

*IPAQ* international PA questionnaire, *BRS* brief resilience scale, *HADS* hospital anxiety and depression scale, *YFAS* yale food addiction scale, *Bcope* Brief-COPE.

The high PA group had greater average BRS resilience scores (p = 0.04) and ability to cope through acceptance of reality (p = 0.04), and a significant difference compared to the low PA group.

Based on PA, there were also significant differences in multiple food addiction measures, as assessed using the Yale Food Addiction Scale (YFAS), with food craving scores being lowest with high PA group. When comparing between high vs. low PA groups, significant differences were found with the following YFAS measures: continued use (p = 0.025), giving up (p = 0.005), time spent (p < 0.001), loss of control (p = 0.01), and symptom count (p < 0.001). Significant differences were also seen between moderate vs. low PA for the following YFAS measures: tolerance (p = 0.04), continued use (p = 0.04), time spent (p = 0.002), loss of control (p = 0.02), and symptom count (p = 0.003; Table 1). There were no differences in macronutrient intake, including energy (kcal), fat (grams), carbohydrate (grams), protein (grams), and cholesterol (mg) when comparing between the PA level groups (Supplemental Table 1).

### PA differentiates brain functional connectivity

**Figure 2:**
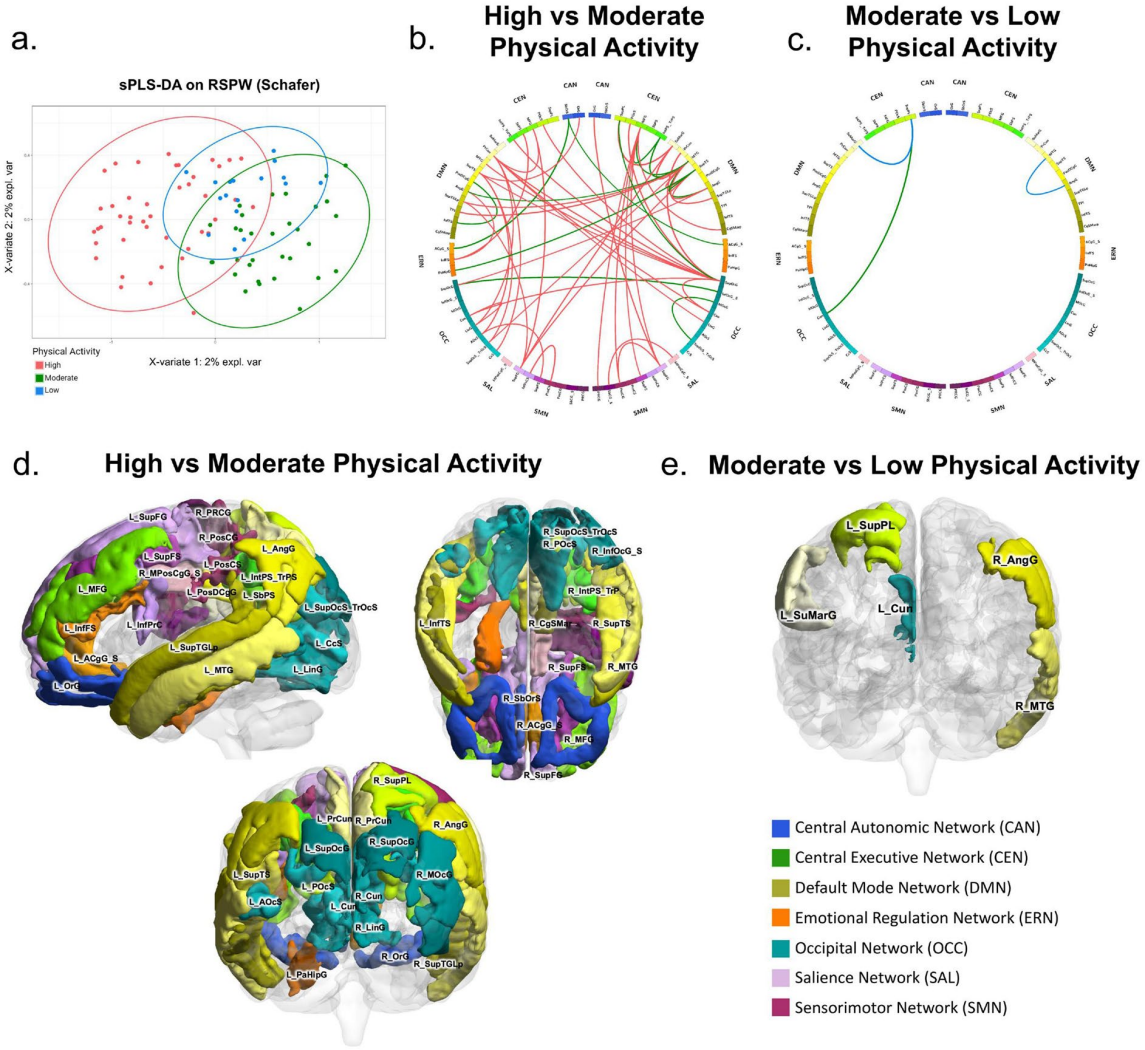
Brain Connectivity Differences Based on Level of PA. (**a**) Clustering plot by SPLS-DA discriminating brain functional connectivity by PA groups. (**b**) Connectogram demonstrating q-value significant (< 0.05) brain connections derived from FDR correction between high vs. moderate PA individuals. Red lines denote increased connectivity in the high group versus green lines represent increased connectivity in the moderate group. (**c**) Connectogram demonstrating q-value significant (< 0.05) brain connections derived from FDR correction between moderate vs. low PA individuals. Green lines denote increased connectivity in the moderate group versus blue lines represent increased connectivity in the low group. (**d**) The q-value significant brain regions when comparing high versus moderate PA are displayed. (**e**) The q-value significant brain regions when comparing moderate versus low PA are displayed.

After adjusting for confounding variables such as age, sex, BMI, and diet, a sPLS-DA of brain functional connectivity displayed significant clustering based on PA level (Fig. 2a). Connectivity between 73 pairs of brain regions were associated with PA. The brain networks involved included the salience (SAL), central autonomic (CAN), central executive (CEN), emotional regulation (ERN), sensorimotor (SMN), default mode (DMN), and occipital (OCC) networks. The specific brain regions are summarized in Table 2.

**Table 2:** Functional Brain Connectivity Differences Based on Physical Activity Levels.

Variable A		Variable B		Loadings	VIP		High vs. moderate	Moderate vs. low	High vs. low
Network	Brain regions	Network	Brain regions		Component 1	Component 2	Interpretation		
Brain component 1									
SAL	R_MPosCgG_S	SMN	R_SbCG_S	− 0.0632	19.1857	17.0948	High ↑	Low ↑	High ↑
CAN	L_OrG	DMN	L_SupTS	− 0.0011	0.3274	0.2917	High ↑	Low ↑	High ↑
CEN	R_SupPL	CEN	R_SupPL	− 0.0378	11.4683	10.2184	High ↑	Low ↑	High ↑
CEN	R_SupPL	DMN	R_SuMarG	− 0.3676	111.6288	99.4632	High ↑	Low ↑	High ↑
CEN	L_SbPS	ERN	L_InfFS	− 0.1571	47.7021	42.5034	High ↑	Low ↑	High ↑
CEN	R_POcS	OCC	R_SupOcG	− 0.2137	64.9121	57.8378	High ↑	Low ↑	High ↑
CEN	L_MFG	SMN	L_SupFG	− 0.0539	16.3791	14.594	High ↑	Low ↑	High ↑
CEN	L_MFG	SMN	L_SupFG	− 0.0282	8.5706	7.6365	High ↑	Low ↑	High ↑
CEN	L_MFG	DMN	L_PrCun	− 0.0029	0.8808	0.7848	High ↑	Low ↑	High ↑
CEN	R_MFG	SMN	R_PRCG	− 0.0112	3.3957	3.0256	High ↑	Low ↑	High ↑
CEN	R_MFG	DMN	R_CgSMarp	− 0.0077	2.351	2.0948	High ↑	Low ↑	High ↑
CEN	R_MFG	SMN	R_PosCG	− 0.0066	2.0067	1.788	High ↑	Low ↑	High ↑
ERN	L_InfFS	DMN	L_AngG	− 0.0143	4.3377	3.8649	High ↑	Low ↑	High ↑
SMN	R_SupFS	CEN	R_MFG	− 0.0313	9.5085	8.4722	High ↑	Low ↑	High ↑
SMN	L_SupFS	OCC	L_AocS	− 0.0894	27.1541	24.1947	High ↑	Low ↑	High ↑
SMN	L_InfPrCS	SMN	L_PosCS	− 0.1311	39.8096	35.471	High ↑	Low ↑	High ↑
SMN	R_SupFG	CEN	R_MFG	− 0.113	34.3084	30.5694	High ↑	Mod ↑	High ↑
SMN	R_SupFG	CAN	R_OrG	− 0.1971	59.8447	53.3226	High ↑	Low ↑	High ↑
SMN	R_SupFG	CAN	R_OrG	− 0.0734	22.3036	19.8729	High ↑	Low ↑	High ↑
SMN	R_SupFG	SMN	R_PosCG	− 0.0676	20.5292	18.2918	High ↑	Low ↑	High ↑
SMN	R_SupFG	CEN	R_MFG	− 0.0313	9.5085	8.4722	High ↑	Low ↑	High ↑
DMN	L_PrCun	OCC	L_LinG	− 0.1247	37.8643	33.7377	High ↑	Low ↑	High ↑
DMN	L_PrCun	OCC	L_CcS	− 0.0778	23.6216	21.0472	High ↑	Low ↑	High ↑
DMN	L_PrCun	OCC	L_LinG	− 0.0804	24.4251	21.7632	High ↑	Low ↑	High ↑
DMN	R_AngG	OCC	L_SupOcG	− 0.0174	5.2722	4.6977	High ↑	Mod ↑	High ↑
DMN	R_PrCun	OCC	R_SupOcG	− 0.3397	103.1674	91.9239	High ↑	Low ↑	High ↑
DMN	R_PrCun	OCC	R_SupOcG	− 0.0691	20.989	18.7016	High ↑	Low ↑	High ↑
DMN	R_PrCun	OCC	R_SupOcG	− 0.0262	7.9594	7.092	High ↑	Low ↑	High ↑
DMN	L_SupTS	OCC	L_AocS	− 0.1596	48.4782	43.1949	High ↑	Low ↑	High ↑
DMN	R_MTG	OCC	L_SupOcS_TrOcS	− 0.0197	5.9976	5.344	High ↑	Low ↑	High ↑
DMN	L_SupTS	OCC	L_SupOcS_TrOcS	− 0.1228	37.2835	33.2202	High ↑	Low ↑	High ↑
DMN	L_SupTGLp	SMN	L_SupFG	− 0.0218	6.6263	5.9042	High ↑	Low ↑	High ↑
DMN	R_SuMarG	SMN	L_SupFG	− 0.1168	35.4697	31.6041	High ↑	Low ↑	High ↑
DMN	R_SupTS	SMN	L_SupFG	− 0.034	10.3189	9.1943	High ↑	Low ↑	High ↑
DMN	R_SupTS	DMN	R_MTG	− 0.1921	58.3395	51.9814	High ↑	Low ↑	High ↑
DMN	R_SuMarG	DMN	R_MTG	− 0.3894	118.2541	105.3663	High ↑	Mod ↑	High ↑
DMN	L_SupTS	DMN	L_TPl	− 0.0598	18.1718	16.1913	High ↑	Low ↑	High ↑
DMN	R_SupTGLp	DMN	R_MTG	− 0.1057	32.1068	28.6077	High ↑	Low ↑	High ↑
DMN	R_SupTS	DMN	R_AngG	− 0.0438	13.3139	11.8629	High ↑	Low ↑	High ↑
DMN	R_MTG	DMN	L_TPl	− 0.1962	59.5757	53.083	High ↑	Low ↑	High ↑
DMN	R_MTG	DMN	L_TPl	− 0.1072	32.5493	29.002	High ↑	Low ↑	High ↑
DMN	R_MTG	DMN	R_SuMarG	− 0.0267	8.1115	7.2275	High ↑	Low ↑	High ↑
DMN	R_MTG	DMN	L_SupTGLp	− 0.0286	8.6991	7.7511	High ↑	Low ↑	High ↑
OCC	R_Cun	DMN	L_PrCun	− 0.0658	19.9697	17.7933	High ↑	Low ↑	High ↑
OCC	R_LinG	DMN	L_PrCun	− 0.027	8.1917	7.2989	High ↑	Low ↑	High ↑
OCC	R_SupOcG	CEN	L_IntPS_TrPS	− 0.1175	35.6806	31.792	High ↑	Low ↑	High ↑
OCC	R_SupOcG	CEN	R_PocS	− 0.0881	26.7565	23.8405	High ↑	Mod ↑	High ↑
OCC	R_SupOcG	DMN	L_PrCun	− 0.1122	34.0694	30.3564	High ↑	Low ↑	High ↑
OCC	R_SupOcG	DMN	L_MTG	− 0.111	33.6996	30.0269	High ↑	Low ↑	High ↑
OCC	R_SupOcG	CEN	R_PocS	− 0.111	33.6996	30.0269	High ↑	Low ↑	High ↑
OCC	R_SupOcG	OCC	R_Cun	− 0.0516	15.6659	13.9586	High ↑	Low ↑	High ↑
OCC	R_SupOcG	CEN	L_PocS	− 0.0168	5.1025	4.5464	High ↑	Low ↑	High ↑
OCC	R_SupOcG	OCC	L_Cun	− 0.0242	7.3584	6.5565	High ↑	Low ↑	High ↑
OCC	R_SupOcG	DMN	L_SupTS	− 0.005	1.5092	1.3447	High ↑	Low ↑	High ↑
OCC	L_Cun	OCC	L_LinG	− 0.047	14.275	12.7192	High ↑	Low ↑	High ↑
CEN	R_MFG	CEN	R_IntPS_TrPS	0.1601	48.6246	43.3253	Mod ↑	Mod ↑	Low ↑
CEN	R_MFG	DMN	R_MTG	0.1456	44.213	39.3946	Mod ↑	Mod ↑	Low ↑
CEN	R_SupPL	OCC	R_SupOcG	0.0287	8.713	7.7634	Mod ↑	Mod ↑	Low ↑
CEN	R_SupPL	OCC	R_SupOcG	0.0131	3.983	3.5489	Mod ↑	Mod ↑	Low ↑
ERN	R_AcgG_S	CAN	L_SbOrS	0.0242	7.36	6.5579	Mod ↑	Low ↑	Low ↑
ERN	L_AcgG_S	CAN	L_SbOrS	0.0912	27.699	24.6803	Mod ↑	Mod ↑	Low ↑
DMN	R_SupTS	DMN	R_SupTS	0.0279	8.4656	7.543	Mod ↑	Mod ↑	Low ↑
DMN	R_SupTS	DMN	R_SupTS	0.0212	6.4297	5.7289	Mod ↑	Mod ↑	Low ↑
DMN	R_MTG	DMN	L_AngG	0.0139	4.223	3.7628	Mod ↑	Low ↑	Low ↑
DMN	R_AngG	DMN	R_MTG	0.116	35.2387	31.3983	v	Mod ↑	Low ↑
DMN	L_PosDCgG	DMN	L_InfTS	0.1524	46.2822	41.2382	Mod ↑	Mod ↑	Low ↑
DMN	R_MTG	CEN	R_IntPS_TrPS	0.2021	61.3663	54.6784	Mod ↑	Mod ↑	Low ↑
DMN	R_SupTS	ERN	L_PaHipG	0.0141	4.286	3.8189	Mod ↑	Mod ↑	Low ↑
OCC	R_MocG	OCC	R_SupOcS_TrOcS	0.075	22.7807	20.2979	Mod ↑	Mod ↑	Low ↑
OCC	R_InfOcG_S	OCC	L_SupOcG	0.1242	37.7238	33.6126	Mod ↑	Mod ↑	Low ↑
Brain component 2									
CEN	L_SupPL	OCC	L_Cun	− 0.948		130.7092	Mod ↑	Mod ↑	High ↑
DMN	R_MTG	DMN	R_AngG	0.2826		38.964	Mod ↑	Low ↑	Low ↑
DMN	L_SuMarG	CEN	L_SupPL	0.1461		20.1381	High ↑	Low ↑	Low ↑

*VIP* variable importance projection.

Comparisons of brain connectivity between each pair of brain regions are made between high vs. moderate, moderate vs. low, and high vs. low PA.

Networks. *SMN* sensorimotor, *DMN* default mode, *SAL* salience, *CEN* central executive, *CAN* central autonomic, *ERN* emotion regulation, *OCC* occipital.

Brain regions: *R_MposCgG_S* Right Middle-posterior part of the cingulate gyrus and sulcus(pMCC), *L_OrG* Left Orbital gyri, *R_SupPL* Right Superior parietal lobule(lateral part of P1), *L_SbPS* Left Subparietal sulcus, *R_PocS* Right Parieto-occipital sulcus(or fissure), *L_MFG* Left Middle frontal gyrus(F2), *R_AcgG_S* Right Anterior part of the cingulate gyrus and sulcus(ACC), *L_AcgG_S* Left Anterior part of the cingulate gyrus and sulcus(ACC), *L_InfFS* Left Inferior frontal sulcus, *R_SupFS* Right Superior frontal sulcus, *L_SupFS* Left Superior frontal sulcus, *L_InfPrCS* Left Inferior part of the precentral sulcus, *R_SupFG* Right Superior frontal gyrus(F1), *L_PrCun* Left Precuneus(medial part of P1), *R_PrCun* Right Precuneus(medial part of P1), *L_SupTS* Left Superior temporal sulcus(parallel sulcus), *R_SupTS* Right Superior temporal sulcus(parallel sulcus), *R_SupTGLp* Right Lateral aspect of the superior temporal gyrus, *R_AngG* Right Angular gyrus, L_PosDCgG Left Posterior-dorsal part of the cingulate gyrus(dPCC), *R_Cun* Right Cuneus(O6), *R_LinG* Right Lingual gyrus, lingual part of the medial occipito-temporal gyrus, (O5), *R_MocG* Right Middle occipital gyrus (O2, lateral occipital gyrus), *R_InfOcG_S* Right Inferior occipital gyrus (O3) and sulcus, *L_SuMarG* Left Supramarginal gyrus, *R_SbCG_S* Right Subcentral gyrus (central operculum) and sulci, *R_SupOcG* Right Superior occipital gyrus (O1), *R_PRCG* Right Precentral gyrus, *R_CgSMarp* Right Marginal branch(or part)of the cingulate sulcus, *L_SbOrS* Left Suborbital sulcus (sulcus rostrales, supraorbital sulcus), *R_MFG* Right Middle frontal gyrus(F2), *L_AocS* Left Anterior occipital sulcus and preoccipital notch(temporo-occipital incisure), *L_PosCS* Left Postcentral sulcus, *R_OrG* Right Orbital gyri, *R_PosCG* Right Postcentral gyrus, *L_CcS* Left Calcarine sulcus, *L_LinG* Left Lingual gyrus, lingual part of the medial occipito-temporal gyrus, (O5), *L_SupOcG* Left Superior occipital gyrus (O1), *L_SupOcS_TrOcS* Left Superior occipital sulcus and transverse occipital sulcus, *L_SupFG* Left Superior frontal gyrus(F1), *R_MTG* Right Middle temporal gyrus(T2), *L_TPl* Left Planum temporale or temporal plane of the superior temporal gyrus, *R_SuMarG* Right Supramarginal gyrus, *L_SupTGLp* Left Lateral aspect of the superior temporal gyrus, *L_AngG* Left Angular gyrus, *L_InfTS* Left Inferior temporal sulcus, *R_IntPS_TrPS* Right Intraparietal sulcus(interparietal sulcus) and transverse parietal sulci, *L_PaHipG* Left Parahippocampal gyrus, parahippocampal part of the medial occipito-temporal gyrus,(T5), *L_IntPS_TrPS* Left Intraparietal sulcus(interparietal sulcus) and transverse parietal sulci, *L_MTG* Left Middle temporal gyrus(T2), *L_PocS* Left Parieto-occipital sulcus(or fissure), *L_Cun* Left Cuneus(O6), *R_SupOcS_TrOcS* Right Superior occipital sulcus and transverse occipital sulcus, *L_SupPL* Left Superior parietal lobule (lateral part of P1).

Compared to both moderate and low PA individuals, those with high PA have increased functional connectivity in 56 pairs of brain connections as summarized in Table 2, involving the DMN, CEN, SMN, OCC, CAN, ERN, and SAL networks (Fig. 2b,d). In contrast to high PA participants, those with moderate and low PA had significantly increased functional connectivity in 16 pairs of brain regions, including brain regions involving the DMN, CEN, OCC, ERN, and CAN networks.

When comparing moderate versus low PA, there were 55 pairs of brain connections that were increased in connectivity in the low PA group, involving the networks DMN, ERN, OCC, CEN, SAL, SMN, and CAN (Fig. 2c,e). Of these brain connections, 51 of these were the same regions that were increased in the high PA group when compared to the moderate and low groups, meaning that these regions were highest in functional connectivity in the high PA group, followed by the low PA individuals, and had the least functional connectivity in the moderate PA group (Table 2).

### PA and gut microbiome composition

**Figure 3:**
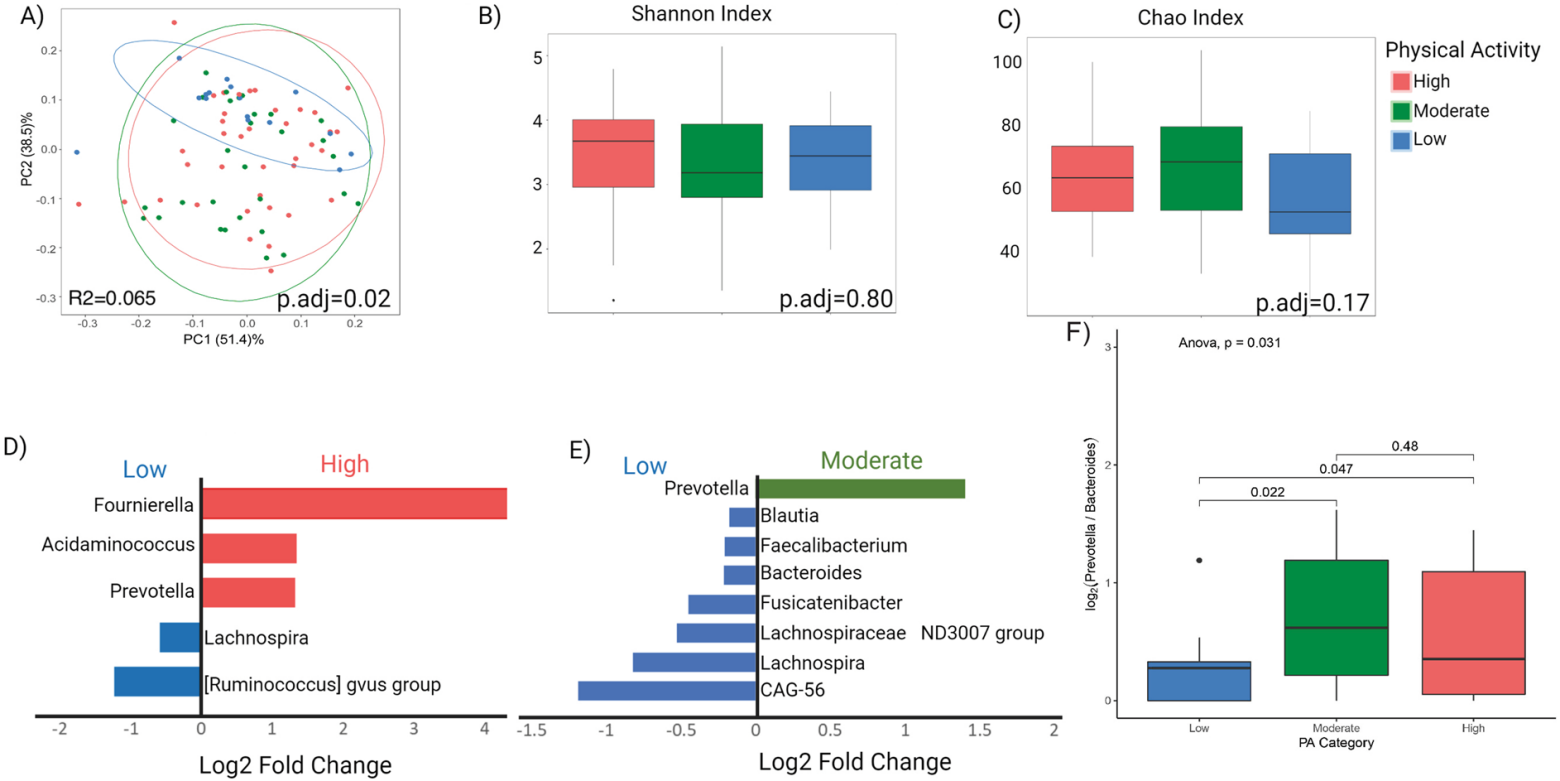
Microbial Taxa Associated with Physical Activity. (**A**) Principal coordinate analysis plot of the microbiome showing beta-diversity by PA level encircled by 95% confidence interval ellipses, adjusting for sex, age, BMI, and diet. (**B**, **C**) Box plot of microbial alpha-diversity by Shannon index and Chao index respectively across PA groups. (**D**) MaAslin2 analysis comparing high vs. low PA showing three genera elevated in high PA participants and two genera elevated in low PA participants. (**E**) MaAslin2 analysis showing one genus increased and seven decreased with moderate when compared to low PA individuals. (**F**) Boxplot depicting the differences in Prevotella to Bacteroides ratio across high, moderate, and low PA groups.

When comparing the three PA groups after adjusting for covariates such as age, sex, BMI and diet, significant differences in beta diversity were seen. The low PA group had a significantly different beta diversity compared to the high and moderate group, which had similar beta diversity signatures (Fig. 3A). No differences were seen with alpha-diversity indices (Fig. 3B,C). Significant differences in relative abundance were also seen when comparing both the high versus low and moderate versus low PA groups with the MaAslin2 analysis, after adjusting for covariates. When comparing high and low PA participants as seen in Fig. 3D, three genera (*Fournierella, Acidaminococcus, and Prevotella*) were higher in abundance and two genera (*Lachnospira, Riminococcus gnavus*) were lower in abundance in the high PA group. *Fournierella* demonstrated the greatest positive fold change when comparing high versus low PA. In the moderate versus low comparison, one genus (*Prevotella*) showed a greater relative abundance and seven genera (*Blautia, Faecalibacterium, Bacteroides, Fusicantenibacter, Lachnospiraceae, Lachnospira, and CAG-56*) had a lower relative abundance in the moderate compared to low PA (Fig. 3E). *Prevotella* showed the greatest positive fold change in the moderate versus low comparison and is increased in relative abundance in a dose-dependent fashion, as it also demonstrated a positive fold change in the high PA group compared to low (Fig. 3D,E).

When comparing Prevotella to Bacteroides ratio, there was overall significant differences seen across all PA groups (p = 0.03). Specifically, there was a significant difference seen between the high vs. low PA groups (p = 0.05) and the moderate vs. low (p = 0.02), but not in the high vs. low (p = 0.48) comparison. Individuals in the moderate PA group had the highest Prevotella to Bacteroides ratio and those in the low PA group had the lowest (Fig. 3F).

Microbial function was assessed by bacterial transcript abundances, which were annotated by KEGG orthology (KO)), and differential abundance testing identified 12 bacterial transcripts that were increased in relative abundance in the low PA group when compared with both the high and low PA groups, which is summarized in Supplemental Fig. 1.

### Fecal metabolites associated with PA

After adjusting for confounding variables such as age, sex, BMI, and diet, 32 metabolites were associated with PA, with 13 categorized as amino acids, seven as lipids, four as nucleotides, three as carbohydrates, two as peptides, two as cofactors, and one as belonging to the energy super pathway (Table 3).

**Table 3:** Fecal metabolites Associated with Physical Activity Levels.

Component 1 metabolites							High vs. mod	Mod vs low	High vs. low
Metabolite	Super pathway	Sub pathway	Loadings		VIP		Interpretation		
			component 1	component 2	component 1	component 2			
Cytosine	Nucleotide	Pyrimidine metabolism, cytidine containing	0.237	0.237	5.6803	3.2177	Mod ↑	Mod ↑	High ↑
Glycosyl Ceramide (D18:2/24:1, D18:1/24:2)	Lipid	Hexosylceramides (HCER)	− 0.1772	0.1772	4.4038	3.059	Mod ↑	Mod ↑	Low ↑
Histidine	Amino Acid	Histidine metabolism	− 0.1663	0.1663	4.184	2.7039	Mod ↑	Mod ↑	High ↑
Docosapentaenoate (n6 DPA; 22:5n6)	Lipid	Hexosylceramides (HCER)	− 0.1437	0.1437	3.7006	2.6406	High ↑	Low ↑	Low ↑
Glycylvaline	Peptide	Dipeptide	− 0.1382	0.1382	3.5839	2.5907	Mod ↑	Mod ↑	High ↑
Tyrosine	Amino Acid	Tyrosine metabolism	− 0.1357	0.1357	3.5245	2.5341	Mod ↑	Mod ↑	High ↑
Proline	Amino Acid	Urea cycle; arginine and proline metabolism	− 0.1307	0.1307	3.4315	2.4611	Mod ↑	Mod ↑	High ↑
Methionine Sulfoxide	Amino Acid	Methionine, cysteine, SAM and taurine metabolism	− 0.129	0.129	3.3683	2.713	Mod ↑	Low ↑	Low ↑
Hypoxanthine	Nucleotide	Purine metabolism, (Hypo)xanthine/inosine containing	0.1289	0.1289	3.3623	2.3695	High ↑	Mod ↑	High ↑
Glycosyl-N-stearoyl-sphingosine (d18:1/18:0)	Lipid	Hexosylceramides (HCER)	− 0.1223	0.1223	3.2429	2.4147	High ↑	Low ↑	L Low ↑
Thymine	Nucleotide	Pyrimidine metabolism, thymine containing	0.1222	0.1222	3.2348	2.3544	High ↑	Mod ↑	High ↑
Homocitrulline	Amino Acid	Urea cycle; arginine and proline metabolism	− 0.1216	0.1216	3.2223	2.4064	Mod ↑	Low ↑	Low ↑
Maltose	Carbohydrate	Glycogen metabolism	0.1202	0.1202	3.1837	2.2996	Mod ↑	Mod ↑	Low ↑
N-Acetyl-Beta-Glucosaminylamine	Carbohydrate	Aminosugar metabolism	− 0.1175	0.1175	3.1472	2.3504	High ↑	Low ↑	High ↑
Ribulose/Xylulose	Carbohydrate	Pentose metabolism	0.1164	0.1164	3.12	2.2495	High ↑	Mod ↑	High ↑
N-Acetyl-1-Methylhistidine	Amino Acid	Histidine metabolism	− 0.1142	0.1142	3.0787	2.4604	Mod ↑	Mod ↑	High ↑
Serine	Amino Acid	Glycine, serine and threonine metabolism	− 0.1141	0.1141	3.0786	2.3618	Mod ↑	Mod ↑	High ↑
Leucine	Amino Acid	Leucine, isoleucine and valine metabolism	− 0.1138	0.1138	3.0722	2.1918	Mod ↑	Mod ↑	High ↑
Arachidoylcarnitine (C20)	Lipid	Fatty acid metabolism (acyl carnitine, long chain saturated)	− 0.1109	0.1109	2.9997	2.1966	Mod ↑	Mod ↑	Low ↑
2'− Deoxyguanosine	Nucleotide	Purine metabolism, guanine containing	− 0.1107	0.1107	3.0064	2.3525	High ↑	Low ↑	Low ↑
Phenylalanine	Amino Acid	Phenylalanine metabolism	− 0.1096	0.1096	2.9798	2.2345	High ↑	Mod ↑	High ↑
Aspartate	Amino Acid	Alanine and aspartate metabolism	− 0.1086	0.1086	2.9638	2.1294	High ↑	Mod ↑	High ↑
Lignoceroylcarnitine (C24)	Lipid	Fatty acid metabolism (acyl carnitine, long chain saturated)	− 0.1061	0.1061	2.9143	2.0832	Mod ↑	Mod ↑	High ↑
Biocytin	Cofactors and Vitamins	Biotin metabolism	− 0.104	0.104	2.8511	2.0857	Mod ↑	Mod ↑	Low ↑
Glycosyl-N-(2-Hydroxynervonoyl)-Sphingosine (D18:1/24:1(2Oh))	Lipid	Hexosylceramides (HCER)	− 0.1038	0.1038	2.8546	2.1456	Mod ↑	Low ↑	Low ↑
Stearoylcarnitine (C18)	Lipid	Fatty acid metabolism (acyl carnitine, long chain saturated)	− 0.1036	0.1036	2.8622	2.0504	High ↑	Low ↑	Low ↑
Glycylisoleucine	Peptide	Dipeptide	− 0.1015	0.1015	2.8063	2.0515	High ↑	Mod ↑	High ↑
Taurolithocholate	Lipid	Secondary bile acid metabolism	− 0.1013	0.1013	2.8077	2.2574	Mod ↑	Mod ↑	Low ↑
Threonine	Amino Acid	Glycine, serine and threonine metabolism	− 0.0985	0.0985	2.7493	2.2574	Mod ↑	Mod ↑	Low ↑

Component 2 metabolites							Interpretation		
Metabolite	Super pathway	Sub pathway	Loadings component 1	Loadings component 2	VIP component 1	VIP component 2	High vs. mod	Mod vs low	High vs. low
Succinate	Energy	TCA cycle		− 0.3954	0.2777	2.853	High ↑	Low ↑	High ↑
1-Methylnicotinamide	Cofactors and vitamins	Nicotinate and nicotinamide metabolism		− 0.3399	0	2.6557	Mod ↑	Mod ↑	Low ↑
Argininate	Amino acid	Urea cycle; arginine and proline metabolism		− 0.3284	1.3653	2.6563	Mod ↑	Mod ↑	High ↑
Tyramine	Amino acid	Tyrosine metabolism		− 0.3079	0.4866	2.5649	High ↑	Low ↑	High ↑
Hyocholate	Lipid	Secondary bile acid metabolism		− 0.2755	0	2.4631	High ↑	Low ↑	Low ↑

*VIP* variable importance projection.

Interpretation column indicates which of the two comparisons had higher values in the groupwise comparisons.

**Figure 4:**
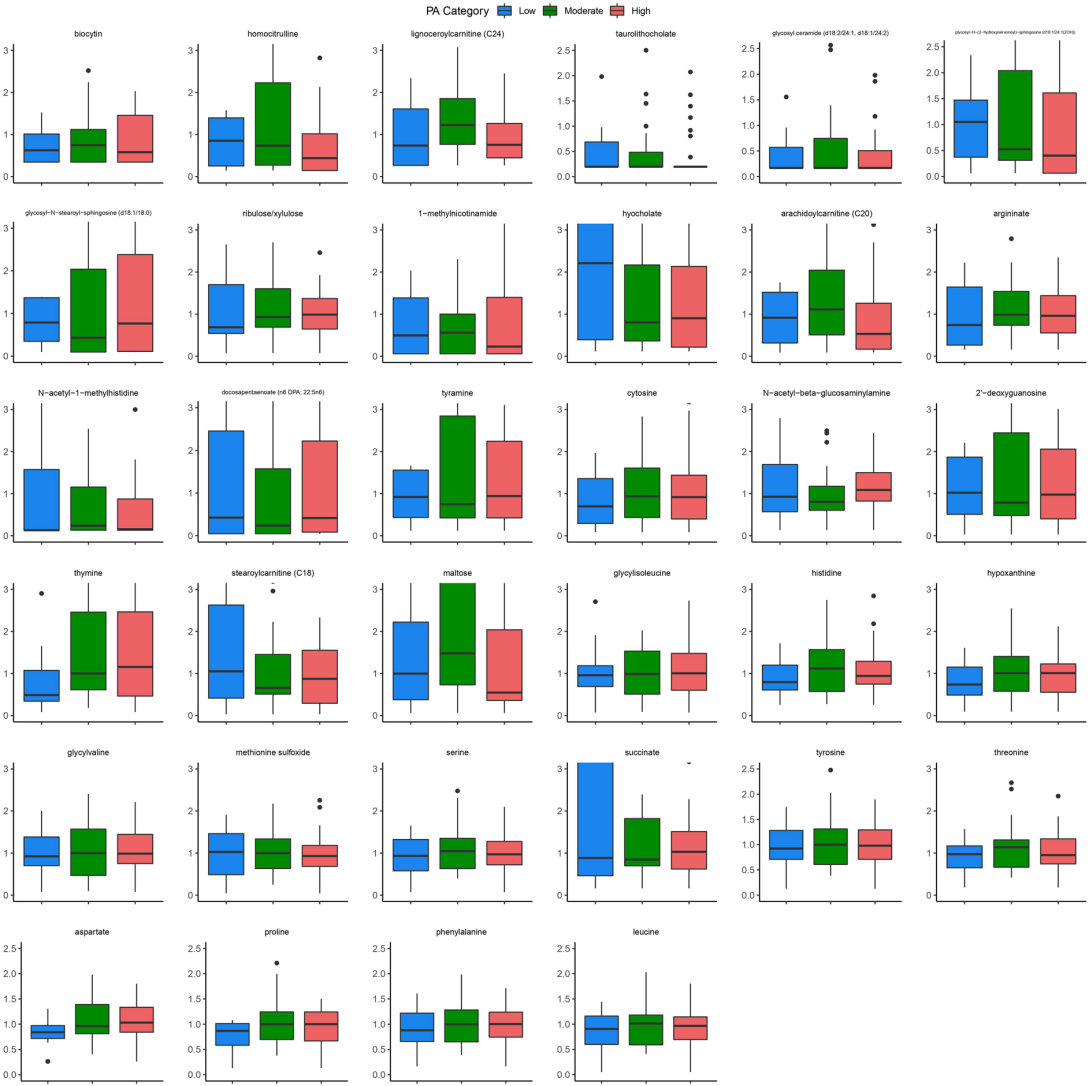
Metabolites Associated with PA. Boxplots depicting the fecal metabolites significantly associated with PA across high, moderate, and low PA groups.

The top three metabolites that were most associated with PA were cytosine, glycosyl ceramide (D18:2/24:1, D18:1/24:2), and histidine. Additionally, lignoceroylcarnitine (C24) levels were highest in the moderate PA group and also elevated in the high PA group when compared to the low PA group. Glycosyl-N-(2-hydroxynervonoyl)sphingosine (D18:1/24:1(2Oh)) and 1-methylnicotinamide showed a dose dependent negative trend with more PA, with highest levels in the low PA group and lowest in those with high PA. On the other hand, ribulose, phenylalanine, aspartate, thymine, hypoxanthine, and glycylisoleucine levels showed a positive trend with PA, with highest levels in the high PA group. A summary of the trends for each metabolite that were associated with PA is shown in Fig. 4.

Significant associations were also identified between the significant psychosocial variables (coping, resilience score, food addiction measures, education), the metabolites, and pairs of connected brain regions across all physical activity group comparisons, which is summarized in Table 4.

**Table 4:** Physical Activity Interacts with Psychosocial Variables, Gut Microbiome, Fecal Metabolites, and Brain Connectivity.

High physical activity vs. low physical activity					
Brain vs Clinical					
Variable 1	Variable 2	r	p	p-adjusted	FDR
DMN R_SupTS DMN R_SupTS	BCope_Acceptance	− 0.4649	0.0006	0.0233	0.0260
DMN R_MTG OCC L_SupOcS_TrOcS	BCope_Acceptance	0.3658	0.0083	0.0397	0.0377
ERN L_InfFS DMN L_AngG	YFAS_ContinuedUse	− 0.3752	0.0061	0.0375	0.0359
DMN R_SupTS DMN R_MTG	YFAS_ContinuedUse	− 0.3679	0.0073	0.0394	0.0362
DMN R_SupTS DMN R_MTG	YFAS_SymptomCount	− 0.3889	0.0044	0.0375	0.0339
SMN L_SupFS OCC L_AOcS	YFAS_SymptomCount	− 0.3489	0.0112	0.0448	0.0411
DMN L_PosDCgG DMN L_InfTS	YFAS_TimeSpent	0.3862	0.0047	0.0375	0.0339
DMN L_PrCun OCC L_LinG	YFAS_TimeSpent	− 0.3664	0.0075	0.0397	0.0362
DMN R_MTG DMN L_AngG	YFAS_TimeSpent	0.3599	0.0088	0.0397	0.0377
DMN R_SupTS DMN R_MTG	YFAS_TimeSpent	− 0.3322	0.0161	0.0488	0.0443

Brain vs. metabolite					
Variable 1	Variable 1	r	p	p-adjusted	FDR
DMN R_MTG CEN R_IntPS_TrPS	1-methylnicotinamide	0.5053	0.0001	0.0102	0.0251
SMN L_InfPrCS SMN L_PosCS	2'-deoxyguanosine	− 0.3447	0.0123	0.0475	0.0425
CAN L_OrG DMN L_SupTS	Arachidoylcarnitine (C20)	0.3807	0.0054	0.0375	0.0354
CEN R_MFG SMN R_PosCG	Arachidoylcarnitine (C20)	− 0.3717	0.0067	0.0386	0.0362
DMN L_PrCun OCC L_CcS	Arachidoylcarnitine (C20)	− 0.3393	0.0139	0.0488	0.0443
CEN R_SupPL DMN R_SuMarG	Biocytin	0.4084	0.0026	0.0334	0.0316
DMN L_PosDCgG DMN L_InfTS	Biocytin	− 0.3826	0.0051	0.0375	0.0353
SMN L_InfPrCS SMN L_PosCS	Biocytin	− 0.3637	0.0080	0.0397	0.0376
DMN R_SuMarG DMN R_MTG	Cytosine	− 0.4060	0.0028	0.0334	0.0316
DMN R_SupTS SMN L_SupFG	Cytosine	− 0.4027	0.0031	0.0334	0.0316
DMN R_MTG DMN L_TPl	Cytosine	− 0.3769	0.0059	0.0375	0.0359
SMN R_SupFG CAN R_OrG	Cytosine	− 0.3729	0.0065	0.0383	0.0362
DMN R_AngG DMN R_MTG	Docosapentaenoate (n6 DPA; 22:5n6)	0.3703	0.0069	0.0389	0.0362
DMN R_MTG OCC L_SupOcS_TrOcS	glycosyl ceramide (d18:2/24:1, d18:1/24:2)	0.3499	0.0110	0.0446	0.0410
OCC R_SupOcG CEN L_POcS	glycosyl-N-(2-hydroxynervonoyl)-sphingosine (d18:1/24:1(2OH))	− 0.3361	0.0149	0.0488	0.0443
SAL R_MPosCgG_S SMN R_SbCG_S	glycosyl-N-stearoyl-sphingosine (d18:1/18:0)	0.3352	0.0151	0.0488	0.0443
DMN L_PosDCgG DMN L_InfTS	Glycylisoleucine	− 0.3842	0.0049	0.0375	0.0348
DMN R_AngG OCC L_SupOcG	Glycylisoleucine	0.3380	0.0143	0.0488	0.0443
DMN L_PosDCgG DMN L_InfTS	Glycylvaline	− 0.3338	0.0156	0.0488	0.0443
CEN R_SupPL DMN R_SuMarG	Histidine	0.3607	0.0086	0.0397	0.0377
DMN R_SuMarG DMN R_MTG	Homocitrulline	− 0.3873	0.0046	0.0375	0.0339
DMN R_MTG DMN R_SuMarG	Homocitrulline	− 0.3589	0.0090	0.0397	0.0377
CEN R_MFG SMN R_PosCG	Hyocholate	− 0.3813	0.0053	0.0375	0.0354
OCC R_SupOcG OCC R_Cun	Hypoxanthine	0.3759	0.0060	0.0375	0.0359
CEN R_SupPL DMN R_SuMarG	Leucine	0.3786	0.0056	0.0375	0.0359
CEN L_SupPL OCC L_Cun	Lignoceroylcarnitine (C24)	0.4082	0.0027	0.0334	0.0316
CEN R_SupPL DMN R_SuMarG	Lignoceroylcarnitine (C24)	0.3321	0.0162	0.0488	0.0443
SMN R_SupFG CAN R_OrG	Maltose	− 0.3334	0.0157	0.0488	0.0443
CEN R_MFG SMN R_PRCG	N-acetyl-beta-glucosaminylamine	− 0.3570	0.0094	0.0397	0.0377
CEN R_SupPL DMN R_SuMarG	Phenylalanine	0.3654	0.0077	0.0397	0.0365
DMN R_SupTS SMN L_SupFG	Phenylalanine	− 0.3367	0.0147	0.0488	0.0443
CEN R_SupPL DMN R_SuMarG	Proline	0.4369	0.0012	0.0283	0.0260
CEN R_SupPL DMN R_SuMarG	Serine	0.3749	0.0062	0.0375	0.0359
CEN R_MFG SMN R_PosCG	Stearoylcarnitine (C18)	− 0.4473	0.0009	0.0269	0.0260
OCC R_MOcG OCC R_SupOcS_TrOcS	Succinate	0.3886	0.0044	0.0375	0.0339
CEN R_SupPL OCC R_SupOcG	Succinate	0.3434	0.0127	0.0482	0.0433
DMN L_SupTS DMN L_TPl	Succinate	0.3405	0.0135	0.0488	0.0441
CAN L_OrG DMN L_SupTS	Taurolithocholate	0.3565	0.0095	0.0397	0.0377
CEN R_SupPL DMN R_SuMarG	Threonine	0.4021	0.0031	0.0334	0.0316
DMN L_SupTS DMN L_TPl	Tyramine	0.4363	0.0012	0.0283	0.0260
CEN R_SupPL OCC R_SupOcG	Tyramine	0.4205	0.0019	0.0332	0.0302
DMN R_SupTS SMN L_SupFG	Tyrosine	− 0.3593	0.0089	0.0397	0.0377
CEN R_SupPL DMN R_SuMarG	Tyrosine	0.3449	0.0123	0.0475	0.0425

Brain vs. microbiome					
Variable 1	Variable 2	r	p	p-adjusted	FDR
DMN L_PrCun OCC L_CcS	[Ruminococcus] gvus group	− 0.3788	0.0056	0.0375	0.0359
SAL R_MPosCgG_S SMN R_SbCG_S	[Ruminococcus] gvus group	− 0.3527	0.0103	0.0426	0.0400
OCC R_LinG DMN L_PrCun	[Ruminococcus] gvus group	− 0.3408	0.0134	0.0488	0.0440
DMN R_MTG DMN L_TPl	Acidaminococcus	− 0.3576	0.0093	0.0397	0.0377
DMN R_MTG DMN L_TPl	Fournierella	0.4342	0.0013	0.0283	0.0260
DMN R_MTG DMN L_AngG	Fournierella	− 0.4017	0.0032	0.0334	0.0316
DMN R_MTG DMN L_TPl	Fournierella	0.3567	0.0094	0.0397	0.0377
DMN R_MTG CEN R_IntPS_TrPS	Fournierella	− 0.3318	0.0163	0.0488	0.0443
SMN R_SupFG CAN R_OrG	Lachnospira	− 0.3685	0.0072	0.0394	0.0362
CEN R_MFG DMN R_MTG	Lachnospira	0.3597	0.0088	0.0397	0.0377
DMN R_PrCun OCC R_SupOcG	Prevotella	0.4317	0.0014	0.0283	0.0270
OCC R_SupOcG DMN L_MTG	Prevotella	0.3410	0.0134	0.0488	0.0440

Metabolite vs clinical					
Variable 1	Variable 2	r	p	p-adjusted	FDR
Tyramine	BCope_Acceptance	− 0.4135	0.0023	0.0334	0.0316
Succinate	BCope_Acceptance	− 0.3952	0.0037	0.0367	0.0324
Succinate	YFAS_GivenUp	0.4117	0.0024	0.0334	0.0316
Succinate	YFAS_LossControl	0.5653	0.0000	0.0015	0.0038
Succinate	YFAS_TimeSpent	0.3911	0.0038	0.0367	0.0324

Metabolite vs microbiome					
Variable 1	Variable 2	r	p	p-adjusted	FDR
Hyocholate	[Ruminococcus] gvus group	0.4903	0.0002	0.0102	0.0251
N-acetyl-1-methylhistidine	Lachnospira	0.5745	0.0000	0.0014	0.0033
taurolithocholate	Lachnospira	0.4645	0.0004	0.0196	0.0260
Hyocholate	Lachnospira	0.4030	0.0025	0.0334	0.0316
Stearoylcarnitine (C18)	Lachnospira	0.3842	0.0041	0.0375	0.0335
Tyramine	Prevotella	0.4484	0.0007	0.0233	0.0260
Ribulose/xylulose	Prevotella	0.4140	0.0019	0.0332	0.0301

Moderate physical activity vs. low physical activity					
Brain vs clinical					
Variable 1	Variable 2	r	p	p-adjusted	FDR
CEN L_MFG DMN L_PrCun	YFAS_ContinuedUse	0.4843	0.0007	0.0202	0.0260
DMN R_SupTS DMN R_AngG	YFAS_LossControl	0.3605	0.0150	0.0370	0.0443
CEN L_MFG DMN L_PrCun	YFAS_SymptomCount	0.4261	0.0035	0.0276	0.0319
DMN R_SupTS SMN L_SupFG	YFAS_TimeSpent	0.4275	0.0034	0.0276	0.0319
DMN R_SupTS DMN R_AngG	YFAS_TimeSpent	0.3823	0.0096	0.0334	0.0377

Brain vs metabolites					
Variable 1	Variable 2	r	p	p-adjusted	FDR
OCC R_SupOcG CEN R_POcS	1-methylnicotinamide	− 0.3612	0.0148	0.0370	0.0443
OCC R_MOcG OCC R_SupOcS_TrOcS	1-methylnicotinamide	0.3605	0.0150	0.0370	0.0443
SMN R_SupFG CAN R_OrG	Argininate	− 0.3932	0.0075	0.0325	0.0362
DMN R_SupTS ERN L_PaHipG	Argininate	− 0.3646	0.0138	0.0370	0.0443
DMN R_SupTGLp DMN R_MTG	Argininate	− 0.3580	0.0158	0.0370	0.0443
DMN R_MTG DMN L_SupTGLp	Argininate	− 0.3562	0.0163	0.0374	0.0443
DMN R_MTG DMN L_SupTGLp	Aspartate	− 0.3716	0.0120	0.0369	0.0425
OCC R_SupOcG DMN L_PrCun	Biocytin	0.3889	0.0083	0.0334	0.0377
OCC R_Cun DMN L_PrCun	Biocytin	0.3636	0.0141	0.0370	0.0443
DMN L_SupTS OCC L_SupOcS_TrOcS	Glycosyl ceramide (d18:2/24:1, d18:1/24:2)	0.3980	0.0068	0.0325	0.0362
CEN L_MFG SMN L_SupFG	Glycosyl ceramide (d18:2/24:1, d18:1/24:2)	0.3863	0.0088	0.0334	0.0377
DMN R_MTG DMN L_TPl	Glycylisoleucine	− 0.4878	0.0007	0.0202	0.0260
OCC R_Cun DMN L_PrCun	Glycylisoleucine	0.4829	0.0008	0.0202	0.0260
DMN R_SuMarG DMN R_MTG	Glycylisoleucine	− 0.4400	0.0025	0.0274	0.0316
OCC R_SupOcG DMN L_PrCun	Glycylisoleucine	0.4220	0.0039	0.0285	0.0324
DMN R_MTG DMN R_SuMarG	Glycylisoleucine	− 0.3878	0.0085	0.0334	0.0377
DMN R_MTG DMN L_TPl	Glycylisoleucine	− 0.3633	0.0142	0.0370	0.0443
OCC R_SupOcG DMN L_PrCun	Glycylvaline	0.4727	0.0010	0.0202	0.0260
OCC R_Cun DMN L_PrCun	Glycylvaline	0.4697	0.0011	0.0202	0.0260
DMN R_MTG DMN L_SupTGLp	Glycylvaline	− 0.4268	0.0035	0.0276	0.0319
DMN R_SuMarG DMN R_MTG	Glycylvaline	− 0.4159	0.0045	0.0294	0.0339
DMN R_MTG DMN L_TPl	Glycylvaline	− 0.3950	0.0073	0.0325	0.0362
OCC R_SupOcG CEN R_POcS	Histidine	0.4171	0.0044	0.0294	0.0339
DMN R_SupTS DMN R_MTG	Histidine	− 0.3938	0.0074	0.0325	0.0362
DMN R_MTG CEN R_IntPS_TrPS	Histidine	0.3669	0.0132	0.0370	0.0440
DMN R_MTG DMN L_SupTGLp	Histidine	− 0.3666	0.0133	0.0370	0.0440
SMN R_SupFG CEN R_MFG	Histidine	− 0.3573	0.0160	0.0370	0.0443
OCC R_MOcG OCC R_SupOcS_TrOcS	Hyocholate	0.4026	0.0061	0.0325	0.0359
DMN R_SuMarG DMN R_MTG	Hyocholate	-0.3669	0.0132	0.0370	0.0440
OCC R_SupOcG DMN L_PrCun	Leucine	0.4539	0.0017	0.0232	0.0301
DMN R_MTG DMN L_SupTGLp	Leucine	− 0.4294	0.0032	0.0276	0.0319
OCC R_Cun DMN L_PrCun	Leucine	0.3941	0.0074	0.0325	0.0362
DMN R_MTG DMN L_TPl	Leucine	− 0.3600	0.0151	0.0370	0.0443
SMN R_SupFG CAN R_OrG	Maltose	− 0.3576	0.0159	0.0370	0.0443
OCC R_SupOcG DMN L_PrCun	Methionine sulfoxide	0.4679	0.0012	0.0202	0.0260
OCC R_Cun DMN L_PrCun	Methionine sulfoxide	0.3989	0.0066	0.0325	0.0362
OCC R_Cun DMN L_PrCun	N-acetyl-beta-glucosaminylamine	0.4008	0.0064	0.0325	0.0362
DMN R_MTG DMN L_SupTGLp	Phenylalanine	− 0.4547	0.0017	0.0232	0.0301
OCC R_SupOcG DMN L_PrCun	Phenylalanine	0.4317	0.0031	0.0276	0.0316
DMN R_SuMarG DMN R_MTG	Phenylalanine	− 0.4282	0.0033	0.0276	0.0319
DMN R_MTG DMN L_TPl	Phenylalanine	− 0.4126	0.0049	0.0297	0.0347
OCC R_Cun DMN L_PrCun	Phenylalanine	0.3852	0.0090	0.0334	0.0377
DMN R_SupTS DMN R_AngG	Phenylalanine	− 0.3754	0.0110	0.0363	0.0410
DMN R_MTG DMN L_SupTGLp	Proline	− 0.3712	0.0121	0.0369	0.0425
CEN R_SupPL OCC R_SupOcG	Ribulose/xylulose	− 0.3840	0.0092	0.0334	0.0377
CEN L_MFG DMN L_PrCun	Ribulose/xylulose	− 0.3585	0.0156	0.0370	0.0443
CEN L_MFG SMN L_SupFG	Ribulose/xylulose	− 0.3578	0.0158	0.0370	0.0443
OCC R_SupOcG DMN L_PrCun	Serine	0.4843	0.0007	0.0202	0.0260
DMN R_MTG DMN L_SupTGLp	Serine	− 0.4353	0.0028	0.0276	0.0316
OCC R_Cun DMN L_PrCun	Serine	0.4142	0.0047	0.0294	0.0339
DMN R_SuMarG DMN R_MTG	Serine	− 0.3663	0.0133	0.0370	0.0440
DMN R_MTG DMN L_TPl	Serine	− 0.3631	0.0142	0.0370	0.0443
CAN L_OrG DMN L_SupTS	Succinate	0.4763	0.0009	0.0202	0.0260
SMN L_SupFS OCC L_AOcS	Succinate	0.3776	0.0106	0.0357	0.0404
DMN R_MTG CEN R_IntPS_TrPS	Taurolithocholate	0.4428	0.0023	0.0268	0.0316
OCC R_SupOcG DMN L_PrCun	threonine	0.4527	0.0018	0.0232	0.0301
DMN R_MTG DMN L_SupTGLp	Threonine	− 0.3840	0.0092	0.0334	0.0377
OCC R_Cun DMN L_PrCun	Threonine	0.3707	0.0122	0.0369	0.0425
CEN R_POcS OCC R_SupOcG	Tyramine	− 0.3959	0.0071	0.0325	0.0362
OCC R_SupOcG DMN L_PrCun	Tyrosine	0.4472	0.0021	0.0253	0.0316
OCC R_Cun DMN L_PrCun	Tyrosine	0.4055	0.0057	0.0325	0.0359
DMN R_MTG DMN L_SupTGLp	Tyrosine	− 0.4046	0.0058	0.0325	0.0359
DMN R_SuMarG DMN R_MTG	Tyrosine	− 0.3795	0.0101	0.0348	0.0395
DMN R_MTG DMN L_TPl	Tyrosine	− 0.3705	0.0122	0.0369	0.0425

Brain vs. microbiome					
Variable 1	Variable 2	r	p	p-adjusted	FDR
DMN L_PosDCgG DMN L_InfTS	Lachnospira	-0.5017	0.0004	0.0202	0.0260
DMN R_MTG DMN L_TPl	Bacteroides	-0.4951	0.0005	0.0202	0.0260
DMN R_SuMarG DMN R_MTG	Bacteroides	-0.4888	0.0007	0.0202	0.0260
DMN R_MTG DMN R_SuMarG	Bacteroides	-0.4803	0.0008	0.0202	0.0260
DMN L_SuMarG CEN L_SupPL	Prevotella	-0.4244	0.0037	0.0278	0.0324
OCC R_SupOcG CEN R_POcS	Lachnospiraceae ND3007 group	0.4199	0.0041	0.0290	0.0335
DMN R_MTG DMN R_SuMarG	Prevotella	0.4108	0.0051	0.0301	0.0353
OCC R_SupOcG CEN R_POcS	Faecalibacterium	0.3979	0.0068	0.0325	0.0362
OCC R_SupOcG OCC R_Cun	Lachnospiraceae ND3007 group	0.3937	0.0075	0.0325	0.0362
SMN L_InfPrCS SMN L_PosCS	Lachnospira	0.3900	0.0081	0.0334	0.0376
DMN R_MTG DMN L_TPl	Bacteroides	− 0.3825	0.0095	0.0334	0.0377
CEN L_MFG SMN L_SupFG	Lachnospira	− 0.3695	0.0125	0.0370	0.0428
OCC R_SupOcG OCC R_Cun	Faecalibacterium	0.3607	0.0149	0.0370	0.0443

Metabolite vs microbiome					
Variable 1	Variable 2	r	p	p-adjusted	FDR
2'-deoxyguanosine	Bacteroides	0.5072	0.0004	0.0202	0.0260
Methionine sulfoxide	Bacteroides	0.4650	0.0013	0.0203	0.0260
Glycylisoleucine	Bacteroides	0.4149	0.0046	0.0294	0.0339
Glycylvaline	Bacteroides	0.3712	0.0121	0.0369	0.0425
N-acetyl-1-methylhistidine	Fusicatenibacter	0.3585	0.0156	0.0370	0.0443
Proline	Lachnospira	− 0.4316	0.0031	0.0276	0.0316
Threonine	Lachnospira	− 0.4034	0.0060	0.0325	0.0359
Leucine	Lachnospira	− 0.3829	0.0094	0.0334	0.0377
Serine	Lachnospira	− 0.3743	0.0113	0.0366	0.0412
Biocytin	Lachnospira	− 0.3621	0.0145	0.0370	0.0443
Methionine sulfoxide	Lachnospira	− 0.3584	0.0156	0.0370	0.0443
Proline	Lachnospiraceae ND3007 group	− 0.3753	0.0111	0.0363	0.0410
Ribulose/xylulose	Prevotella	0.3883	0.0084	0.0334	0.0377
Methionine sulfoxide	Prevotella	− 0.3873	0.0086	0.0334	0.0377

High physical activity vs. moderate physical activity					
Brain vs. clinical					
Variable 1	Variable 2	r	p	p-adjusted	FDR
CEN L_SbPS ERN L_InfFS	Education	-0.4236	0.0006	0.0292	0.0260
CAN L_OrG DMN L_SupTS	Education	-0.3997	0.0013	0.0292	0.0260
DMN L_SupTS OCC L_SupOcS_TrOcS	Education	-0.3715	0.0030	0.0292	0.0316
OCC R_SupOcG DMN L_SupTS	Education	-0.3711	0.0030	0.0292	0.0316
DMN R_SupTS ERN L_PaHipG	Education	0.3661	0.0034	0.0311	0.0319
DMN R_SupTS DMN R_MTG	Education	-0.3360	0.0076	0.0359	0.0362
CEN L_MFG DMN L_PrCun	Education	-0.3225	0.0106	0.0424	0.0404
SMN R_SupFS CEN R_MFG	Education	-0.3203	0.0111	0.0424	0.0410

Brain vs. metabolite					
Variable 1	Variable 2	r	p	p-adjusted	FDR
OCC R_SupOcG CEN L_POcS	1-methylnicotinamide	− 0.3309	0.0071	0.0359	0.0362
CEN R_POcS OCC R_SupOcG	1-methylnicotinamide	− 0.3097	0.0120	0.0443	0.0425
SMN L_InfPrCS SMN L_PosCS	2'-deoxyguanosine	− 0.3019	0.0145	0.0477	0.0443
OCC R_MOcG OCC R_SupOcS_TrOcS	Argininate	0.3790	0.0018	0.0292	0.0301
DMN R_SupTS DMN R_SupTS	Argininate	0.3330	0.0067	0.0359	0.0362
CEN L_SbPS ERN L_InfFS	Argininate	− 0.2967	0.0164	0.0478	0.0443
DMN R_MTG DMN L_SupTGLp	Aspartate	− 0.3551	0.0037	0.0311	0.0324
CEN R_SupPL OCC R_SupOcG	Biocytin	0.3676	0.0026	0.0292	0.0316
DMN R_AngG DMN R_MTG	Docosapentaenoate (n6 DPA; 22:5n6)	0.3380	0.0059	0.0359	0.0359
DMN L_SupTS OCC L_SupOcS_TrOcS	Glycosyl ceramide (d18:2/24:1, d18:1/24:2)	0.3810	0.0017	0.0292	0.0301
DMN R_MTG OCC L_SupOcS_TrOcS	Glycosyl ceramide (d18:2/24:1, d18:1/24:2)	0.3536	0.0039	0.0311	0.0324
DMN L_SupTS OCC L_SupOcS_TrOcS	Glycosyl-N-stearoyl-sphingosine (d18:1/18:0)	0.3506	0.0042	0.0319	0.0335
DMN R_MTG OCC L_SupOcS_TrOcS	Glycosyl-N-stearoyl-sphingosine (d18:1/18:0)	0.3429	0.0052	0.0359	0.0353
OCC R_SupOcG DMN L_PrCun	Glycylisoleucine	0.3283	0.0076	0.0359	0.0362
OCC R_Cun DMN L_PrCun	Glycylisoleucine	0.3220	0.0089	0.0394	0.0377
DMN R_MTG DMN L_SupTGLp	Glycylvaline	− 0.3029	0.0142	0.0477	0.0443
DMN R_SupTS SMN L_SupFG	Histidine	− 0.3072	0.0128	0.0450	0.0434
CEN L_SbPS ERN L_InfFS	Hyocholate	− 0.3401	0.0056	0.0359	0.0359
OCC R_SupOcG CEN L_POcS	Hyocholate	− 0.3133	0.0110	0.0424	0.0410
OCC R_SupOcG OCC R_Cun	Leucine	0.3148	0.0106	0.0424	0.0404
CEN R_SupPL OCC R_SupOcG	Leucine	0.2972	0.0162	0.0478	0.0443
CEN R_MFG CEN R_IntPS_TrPS	Lignoceroylcarnitine (C24)	0.3418	0.0053	0.0359	0.0354
OCC R_SupOcG CEN L_POcS	Maltose	− 0.4012	0.0009	0.0292	0.0260
SMN R_SupFG CAN R_OrG	Maltose	− 0.3948	0.0011	0.0292	0.0260
CEN L_SbPS ERN L_InfFS	Maltose	− 0.3718	0.0023	0.0292	0.0316
CAN L_OrG DMN L_SupTS	Maltose	− 0.3006	0.0150	0.0477	0.0443
OCC R_SupOcG CEN L_IntPS_TrPS	Maltose	− 0.2986	0.0157	0.0477	0.0443
SMN R_SupFG CEN R_MFG	Methionine sulfoxide	− 0.3170	0.0101	0.0424	0.0395
DMN R_MTG DMN L_SupTGLp	N-acetyl-1-methylhistidine	− 0.3743	0.0021	0.0292	0.0316
OCC R_SupOcG CEN L_POcS	N-acetyl-1-methylhistidine	− 0.3254	0.0082	0.0374	0.0377
DMN R_SupTS ERN L_PaHipG	N-acetyl-beta-glucosaminylamine	− 0.3640	0.0029	0.0292	0.0316
CEN R_MFG DMN R_CgSMarp	N-acetyl-beta-glucosaminylamine	0.3639	0.0029	0.0292	0.0316
SMN R_SupFG SMN R_PosCG	N-acetyl-beta-glucosaminylamine	0.3322	0.0069	0.0359	0.0362
DMN R_MTG CEN R_IntPS_TrPS	Taurolithocholate	0.4380	0.0003	0.0292	0.0260
CEN R_SupPL OCC R_SupOcG	Threonine	0.3301	0.0072	0.0359	0.0362
DMN R_MTG DMN L_SupTGLp	Threonine	− 0.3289	0.0075	0.0359	0.0362
OCC R_SupOcG OCC R_Cun	Threonine	0.3089	0.0123	0.0443	0.0425
DMN R_SupTS DMN R_AngG	Threonine	− 0.2992	0.0155	0.0477	0.0443
DMN R_MTG OCC L_SupOcS_TrOcS	Tyramine	− 0.2996	0.0153	0.0477	0.0443

p-value significant < 0.05.

*FDR* false discovery rate.

*BRS* Brief Resilience Scale, *YFAS* Yale Food Addiction Scale, *Bcope* Brief-COPE.

Networks: *SMN* sensorimotor, *DMN* default mode, *SAL* salience, *CEN* central executive, *CAN* central autonomic, *ERN* emotion regulation, *OCC* occipital.

Brain regions: *L_SupPL* Left Superior parietal lobule (lateral part of P1), *L_Cun* Left Cuneus (O6), *R_AngG* Right Angular gyrus, *R_SupTS* Right Superior temporal sulcus (parallel sulcus), *R_MTG* Right Middle temporal gyrus (T2), *L_SupTS* Left Superior temporal sulcus (parallel sulcus), *L_AocS* Left Anterior occipital sulcus and preoccipital notch (temporo-occipital incisure), *L_SbPS* Left Subparietal sulcus, *L_InfFS* Left Inferior frontal sulcus, *R_SupFS* Right Superior frontal sulcus, *R_MFG* Right Middle frontal gyrus(F2), *L_InfFS* Left Inferior frontal sulcus, *L_AngG* Left Angular gyrus, L_PaHipG Left Parahippocampal gyrus, parahippocampal part of the medial occipito-temporal gyrus,(T5), *L_OrG* Left Orbital gyri, *R_SuMarG* Right Supramarginal gyrus, R_SupPL Right Superior parietal lobule(lateral part of P1), *L_TPl* Left Planum temporal or temporal plane of the superior temporal gyrus, *L_PosDCgG* Left Posterior-dorsal part of the cingulate gyrus(dPCC), *L_InfTS* Left Inferior temporal sulcus, *R_MFG* Right Middle frontal gyrus(F2), *L_InfPrCS* Left Inferior part of the precentral sulcus, *L_PosCS* Left Postcentral sulcus, *L_LinG* Left Lingual gyrus, lingual part of the medial occipito-temporal gyrus, (O5), *L_SupOcS_TrOcS* Left Superior occipital sulcus and transverse occipital sulcus, *L_IntPS_TrPS* Left Intraparietal sulcus(interparietal sulcus) and transverse parietal sulci, *R_AngG* Right Angular gyrus, *R_SupOcG* Right Superior occipital gyrus (O1), *L_PrCun* Left Precuneus(medial part of P1), *L_MTG* Left Middle temporal gyrus(T2), *R_SupTGLp* Right Lateral aspect of the superior temporal gyrus, *L_SbOrS* Left Suborbital sulcus (sulcus rostrales, supraorbital sulcus), *L_AcgG_S* Left Anterior part of the cingulate gyrus and sulcus(ACC), *L_PrCun* Left Precuneus(medial part of P1), *L_CcS* Left Calcarine sulcus, *R_SupOcS_TrOcS* Right Superior occipital sulcus and transverse occipital sulcus, *R_MocG* Right Middle occipital gyrus (O2, lateral occipital gyrus), *R_OrG* Right Orbital gyri, *R_SupFG* Right Superior frontal gyrus(F1), *R_PrCun* Right Precuneus (medial part of P1), *R_SupOcG* Right Superior occipital gyrus (O1), *R_Cun* Right Cuneus(O6), *R_MposCgG_S* Right Middle-posterior part of the cingulate gyrus and sulcus(pMCC), *R_SbCG_S* Right Subcentral gyrus (central operculum) and sulci, *L_MFG* Left Middle frontal gyrus(F2), *L_SupTGLp* Left Lateral aspect of the superior temporal gyrus, *L_SupFG* Left Superior frontal gyrus(F1), *R_MTG* Right Middle temporal gyrus(T2), *R_PRCG* Right Precentral gyrus, *R_CgSMarp* Right Marginal branch(or part)of the cingulate sulcus, *R_PosCG* Right Postcentral gyrus, *L_SupTS* Left Superior temporal sulcus(parallel sulcus).

## Discussion

In this study, we demonstrated that there are significant alterations associated with PA seen in the functional connectivity of the brain, beta diversity and relative abundance of the gut microbiome, and metabolites produced. These BGM system alterations are associated with improved psychosocial measures in an overweight and obese population. Given that individuals with high BMI face additional weight-related stressors compared to normal-weight individuals, these findings explore the possible utility of PA in preventing and treating mental illnesses in the high BMI population and how PA possibly promotes health beyond solely metabolic regulation^3,42^.

In this study, an association was identified between higher PA and greater resilience, which is a known protective factor against the development of psychiatric disorders such as depression and post-traumatic stress disorder (PTSD)^43^. This finding is consistent with previous studies showing higher resilience scores in participants with more physical exercise in various populations^44–46^. Within many of the YFAS food addiction measures, low PA was associated with the highest food addiction scores while high PA participants had the lowest scores, and these findings were associated with altered connectivity within brain regions of the DMN. Specifically, the moderate PA participants when compared to those with high PA had increased connectivity between the angular gyrus and middle temporal gyrus regions, which a previous study demonstrated to be increased in activation when participants passively viewed visual food cues versus while they actively inhibited the urge to eat^47^. Previous studies on the chronic effects of exercise on appetite parameters have been largely conflicting, with some studies reporting an increase in subjective appetite in the fasted state after aerobic exercise, whereas others have reported a reduction or no change^48–51^. Our findings suggest that the subjective appetite responses to PA may be intensity-dependent, with a greater amount of PA associated with reduced appetite.

Several of the significant microbiome genera found to be associated with PA have previously been studied in the context of psychiatric illnesses. Specifically, individuals with lower PA were seen to have increased relative abundance of *Blautia* and *Bacteroides*, which have both been shown to be increased in patients with major depressive disorder (MDD) and bipolar disorder^52,53^. *Prevotella* has also been shown to be decreased in patients with MDD, and we saw that overall higher PA was associated with a positive fold change with *Prevotella* in both the high versus low and moderate versus low comparisons^52^. When looking at past studies involving patients with general anxiety disorder, higher levels of *Bacteroides* and lower levels of *Prevotella* correlated with severity of anxiety^54,55^. Therefore, the significantly increased Prevotella to Bacteroides ratio that we observed in both the moderate and high PA groups in comparison to the low PA group may suggest that increased physical activity is associated with microbiome signatures protective against depression and anxiety. In addition, we found in our study that low PA was associated with *Ruminococcus gnavus*, which can degrade mucins and lead to gut permeability^56^. There is an extensive body of data that has shown that depression is associated with a low-grade intestinal inflammation, which may allow invasive bacteria to translocation into the systemic circulation^57,58^. This can then trigger an increase in plasma immunoglobulins targeting these bacteria and could explain why clinical depression is accompanied by increases in IgA and/or IgM^59^. Overall, these findings suggest that with more PA, individuals with higher BMI can encourage a microbiome signature that is protective against developing certain psychiatric illnesses such as depression and anxiety.

In addition to *Prevotella’s* association with psychiatric disorders, it is known to predict increased weight loss in overweight individuals and is linked with dietary fiber induced improvements in glucose metabolism through increasing fasting plasma insulin^60,61^. As consistent with previous findings, higher *Prevotella* was associated with more PA when comparing high versus low PA individuals, further demonstrating the crucial role of PA in metabolic and weight regulation through alterations in the microbiome^62^. We also observed that *Fournierella* had the greatest increase in relative abundance in the high PA versus the low PA group. In a population of participants with abdominal obesity, *Fournierella* has been found to be positively associated with a green-Mediterranean diet as well as reduced intrahepatic fat overtime^63^. The increased abundance of these genera associated with lean-phenotypes and reduced intrahepatic fat further illustrate a widely accepted finding that more PA promotes a metabolically healthy microbiome that may prevent further weight gain in individuals who already have higher BMI.

Previous studies have also studied some of the metabolites that we found associated with PA in the context of cognitive health. In our study, we observed a negative trend between the metabolite glycosyl-N-(2-hydroxynervonoyl)-sphingosine (d18: 1/24: 1(2OH)) and increased PA. In a Puerto Rican study evaluating metabolites associated with cognitive function in a non-diabetic population, glycosyl-N-(2-hydroxynervonoyl)-sphingosine (d18: 1/24: 1(2OH)) was found to be related to poor cognition, with participants that scored higher on cognitive function having lower levels of glycosyl-N-(2-hydroxynervonoyl)-sphingosine (d18: 1/24: 1(2OH))^64^. Additionally, we found that histidine levels were higher in the high and moderate PA groups when compared to the low PA group. Studies have shown that histidine intake improves cognitive function, potentially via the metabolism of histidine to histamine, and the histamine receptors (H1 and H3) in the brain are involved in functions related to anxiety, stress, appetite, and sleep^65^. This suggests that increased PA potentially could contribute to promoting better cognitive function and mental health via influence on the metabolites.

PA -associated differences were also seen in brain functional connectivity, most notably when comparing the high versus moderate PA groups. The superior frontal gyrus (SupFG) and middle frontal gyrus (MFG), which are both frontal lobe regions implicated in general inhibitory control but also appetite control, were increased in connectivity in the high PA group compared to moderate PA, which is consistent with the clinical findings of lowest food cravings with high PA and highest food cravings with moderate PA^66^. A possible explanation is that the MFG has also been proposed to act as a circuit-breaker between the ventral and dorsal attention networks, and thus allows for top-down reorientation of attention from endogenous stimuli such as hunger cues to exogenous stimuli in the environment^67^. Similar to our findings, other studies have demonstrated that in comparison to obese individuals, previously obese individuals who successfully maintained weight loss as well as lean individuals have greater activation in the SupFG in response to food cues and during tasks involving response inhibition^66,68^. SupFG has also been negatively correlated to self-reported impulsivity, with ADHD individuals showing hypoactivity in SupFG and MFG^69^. The finding of increased functional connectivity in the brain between these two regions was also correlated to the increased histidine we observed with PA, which supports the hypothesis that the effects of PA on appetite may be through connections within the gut-brain axis.

In addition, more emotional regulation network regions were increased in connectivity in moderate PA participants, and were linked to central autonomic versus central executive as seen in the high PA group, suggesting more cognitive control over emotional food cravings with more PA. There were also overall more CEN regions increased in connectivity with high PA linked to the somatosensory and default mode networks, in comparison to the increased connectivity between CEN and occipital regions seen in moderate PA individuals. This suggests more cognitive modulation and evaluation of sensory stimuli with high PA, that may contribute to more restraint and less impulsivity in uncontrolled eating.

Our study had several strengths, including the integration of a comprehensive dataset including brain, gut microbiome, fecal metabolite, and psychosocial variables to determine associations with physical activity level. We also utilized consistent sample processing and OTU clustering and considered major covariates in our analyses. However, the directionality and causality between physical activity and alterations in the BGM system cannot be parsed through this study, but cross-sectional studies such as the one we presented here allow for further understanding the role of physical activity in preventing mental illnesses. Future studies including larger and longitudinal and more evenly distributed sample sizes within each physical activity level group are warranted and would allow for more statistical power in the analyses. Including additional serum metabolome markers can also provide further clarity on the metabolites studied. In addition, despite previous studies showing the validity of self-reported physical activity data, the additional use of objective data obtained through accelerometers would enhance the accuracy of the data, which could be explored in future projects^70^.

With the COVID-19 pandemic creating short and long-term mental health consequences in as much as 30% of the general population and individuals already suffering from a psychiatric disease, it is even more crucial to identify evidence-based methods to promote psychological resilience amidst this ongoing global health crisis^71^. We have identified novel targets within the BGM system that may be explored for the prevention and treatment of various psychiatric conditions, which individuals with high BMI are already at higher risk for Ref.^72^. This study will inform the design of future longitudinal studies that will elucidate the directionality of these associations.

## Supplementary Information

Supplementary Figure 1.

Supplementary Information.

## Data Availability

The datasets generated during and/or analyzed during the current study are available from the corresponding author on reasonable request. The raw microbiome sequences can be accessed NIH NCBI BioProject (BioProject ID: PRJNA946906).
