# Supplementary figures and images for: Improved psychosocial measures associated with physical activity may be explained by alterations in brain-gut microbiome signatures
Source: Sci Rep. 2023 Jun 26;13:10332. doi: 10.1038/s41598-023-37009-z (PMC10293244; doi:10.1038/s41598-023-37009-z)

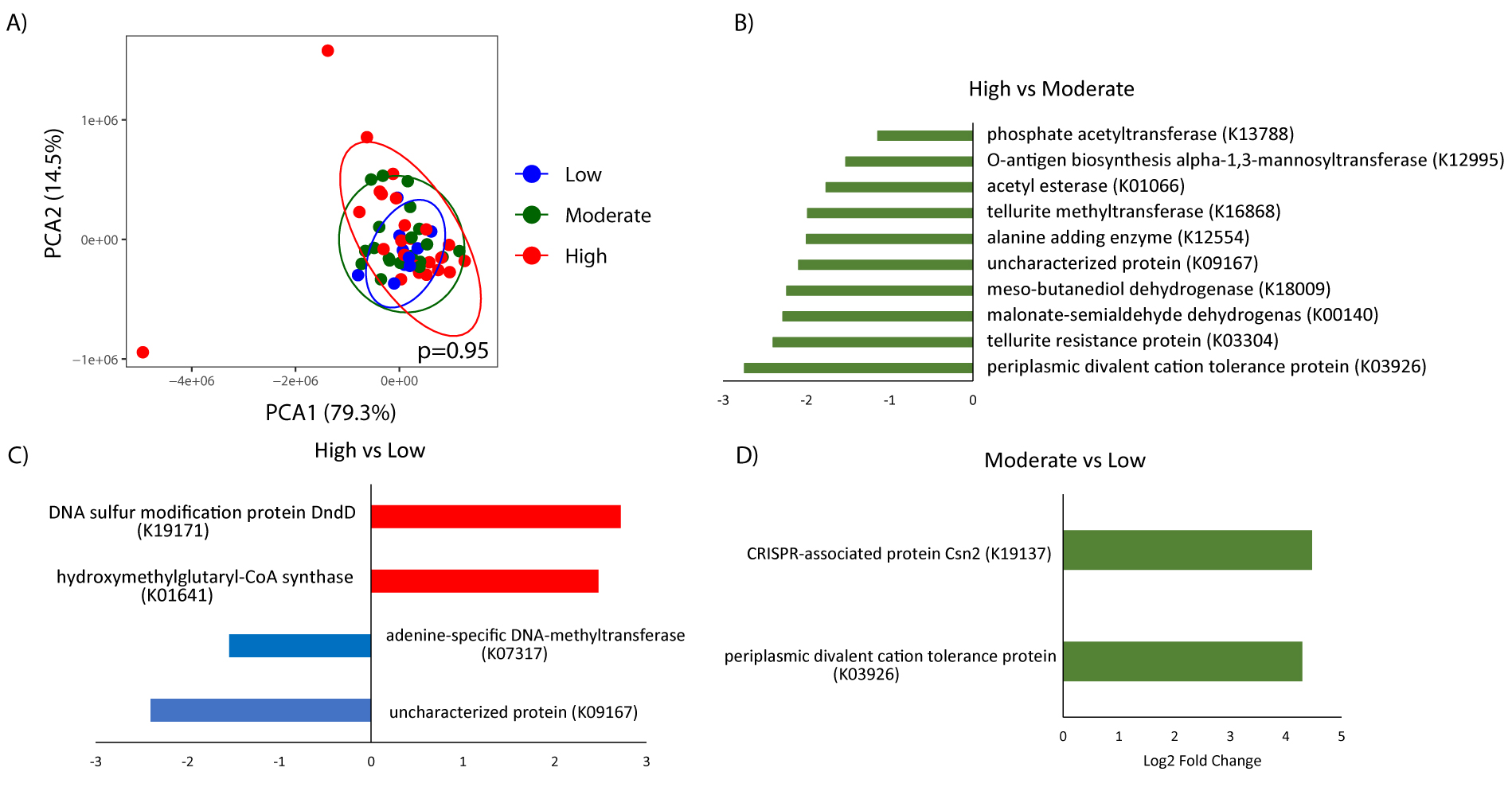

Supplement: Supplementary file 1 — Supplementary Figure 1. [file 41598_2023_37009_MOESM1_ESM.jpg]
