# Supplementary material for: Improved psychosocial measures associated with physical activity may be explained by alterations in brain-gut microbiome signatures
Source: Sci Rep. 2023 Jun 26;13:10332. doi: 10.1038/s41598-023-37009-z (PMC10293244; doi:10.1038/s41598-023-37009-z)
Supplement: Supplementary file 2 — Supplementary Information. [file 41598_2023_37009_MOESM2_ESM.docx]

**Supplemental Table 1: Participant’s Macronutrient Intake Based on Physical Activity Level**

|  | All (N=92) | | | High PA (N=43) | | | Moderate PA (N = 32) | | | Low (N=17) | | | P-Values | | |
| --- | --- | --- | --- | --- | --- | --- | --- | --- | --- | --- | --- | --- | --- | --- | --- |
|  | **Mean** | **SD** | **Range** | **Mean** | **SD** | **Range** | **Mean** | **SD** | **Range** | **Mean** | **SD** | **Range** | **High vs. Mod** | **Mod vs. Low** | **High vs. Low** |
| **ENERGY_KCAL (g)** | 2186.76 | 1459.01 | [ 524.41 10998 ] | 2040.40 | 1185.87 | [ 524.41 6296.5 ] | 2320.43 | 1892.40 | [ 830.85 10998 ] | 2270.15 | 1117.63 | [ 772.44 5224.23 ] | 0.71 | 0.99 | 0.85 |
| **TOTAL_FAT(g)** | 91.14 | 61.36 | [ 19.86 399.36 ] | 86.57 | 50.96 | [ 26.15 238.34 ] | 95.10 | 74.86 | [ 19.86 399.36 ] | 94.10 | 58.32 | [ 25.49 271.06 ] | 0.84 | 1.00 | 0.91 |
| **CARBOHYDRATE (g)** | 254.04 | 187.54 | [ 49.93 1508.69 ] | 230.15 | 147.45 | [ 49.93 783.35 ] | 277.30 | 253.10 | [ 84.13 1508.69 ] | 265.04 | 118.01 | [ 66.55 474.2 ] | 0.56 | 0.97 | 0.80 |
| **PROTEIN (g)** | 92.18 | 64.44 | [ 17.53 367.97 ] | 89.00 | 56.37 | [ 17.53 253.22 ] | 92.53 | 75.78 | [ 20.95 367.97 ] | 98.63 | 62.25 | [ 28.41 286.81 ] | 0.97 | 0.95 | 0.87 |
| **CHOLESTEROL (mg)** | 342.51 | 254.40 | [ 17.13 1366.41 ] | 339.22 | 246.37 | [ 65.1 1115.53 ] | 336.04 | 285.29 | [ 41.31 1366.41 ] | 361.65 | 224.42 | [ 17.13 949.12 ] | 1.00 | 0.94 | 0.95 |

Means and standard deviations are reported for normally distributed data. P-significant <0.05.

**Supplemental Figure 1: Differences in Bacterial Transcript Based on Level of PA**

(a) Clustering plot by SPLS-DA discriminating bacterial transcript by PA groups

(b) Differentially abundant bacterial transcripts, annotated by KEGG KO number and gene name, between high versus moderate PA groups.

(c) Differentially abundant bacterial transcripts, annotated by KEGG KO number and gene name, between high versus low PA groups.

(d) Differentially abundant bacterial transcripts, annotated by KEGG KO number and gene name, between moderate versus low PA groups.
